# Scalable synthesis of a low-cost Zn–MOF with a nonpolar pore surface for efficient separation of methanol-to-olefin products

**DOI:** 10.1039/d5sc03946k

**Published:** 2025-09-16

**Authors:** Yingying Zhang, Suyun Deng, Xingye Cui, Jixiang Yue, Hongliang Huang, Chaozhuang Xue, Huajun Yang, Lei Gan

**Affiliations:** a Jiangsu Key Laboratory of Biomedical Materials, School of Chemistry and Materials Science, Nanjing Normal University Nanjing 210023 China leigan@nnu.edu.cn; b School of Chemistry and Chemical Engineering, Liaocheng University Liaocheng 252059 China; c State Key Laboratory of Separation Membranes and Membrane Processes, School of Chemistry and Chemical Engineering, Tiangong University Tianjin 300387 China

## Abstract

The pursuit of scalable metal–organic framework (MOF) adsorbents for efficient one-step purification of C_2_H_4_ and recovery of C_3_H_6_ holds significant importance for practical applications. It is desirable to incorporate high separation efficiency along with other key norms such as easy scalability, economic feasibility, and eco-friendliness into a single MOF structure. However, this presents a formidable challenge due to the high cost of specially designed ligands, rigorous synthetic conditions, and typically lengthy reaction times. Herein, we present a scalable MOF adsorbent denoted as Zn-hba decorated with a nonpolar pore surface, which successfully integrates all the aforementioned norms. Zn-hba exhibits selective uptake for C_2_H_6_ and C_3_H_6_ over C_2_H_4_ with exceptional selectivity and record-high C_3_H_6_ and exceptional C_2_H_6_ uptake at low pressure, allowing for one-step purification of C_2_H_4_ and recovery of C_3_H_6_ from a ternary mixture of C_2_H_6_/C_2_H_4_/C_3_H_6_. Importantly, Zn-hba is synthesized with a high yield of 42% using commercially available cheap reagents such as zinc acetate, 4-hydroxybenzoic acid, methanol and *n*-amyl alcohol in only 12 hours. The total cost for producing each gram of this adsorbent is as low as $0.14, comparable to that of commercial zeolites and nearly one-tenth the cost of the benchmark MOF MAC-4.

## Abstract 2

This work demonstrates a scalable and cost-effective Zn–MOF *via* facile synthesis for selective uptake of C_2_H_6_ and C_3_H_6_ over C_2_H_4_ with exceptional selectivity, addressing the efficient separation of MTO products.

## Graphical abstract

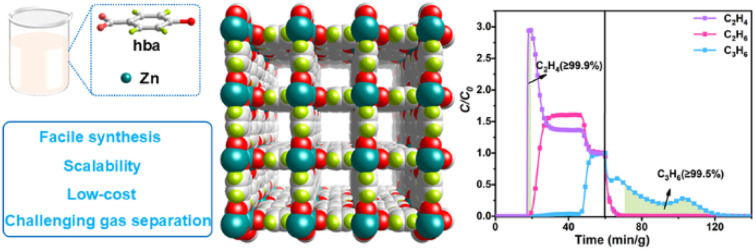

## Introduction

Ethylene (C_2_H_4_) and propylene (C_3_H_6_), primarily produced from hydrocarbon cracking and the methanol-to-olefin (MTO) reaction, are essential feedstocks for the production of downstream chemicals.^1,2^ A crucial step in obtaining high-purity C_2_H_4_ and C_3_H_6_ is the removal of the main impurity C_2_H_6_ and recovery of C_3_H_6_. This is typically achieved through energy-intensive cryogenic cyclic distillation at high pressure, due to their similar physicochemical properties.^3,4^ In the industrial field of polymerization, high-purity C_2_H_4_ and C_3_H_6_ are desirable, with a purity of over 99.9% and 99.5%, respectively. With the growing demand for C_2_H_4_ and C_3_H_6_, exceeding 300 million tons produced in 2023,^5,6^ it is vital to replace the energy-intensive distillation methods with more energy-efficient, cost-effective, and environmentally friendly alternatives. Among these methods, adsorption and separation technology utilizing porous materials holds great promise.^4,7–14^

Metal–organic frameworks (MOFs), as a new class of crystalline porous materials, have been developed for adsorptive separation due to their high modularity, exceptional porosity, and diverse functionality.^3,8,15–18^ To achieve a simplified C_2_H_4_ and C_3_H_6_ purification procedure, the MOF structure must exhibit graded uptake for C_2_H_4_, C_2_H_6_ and C_3_H_6_, with high selectivity and capture capability. Most MOFs have been reported to preferentially adsorb C_2_H_4_ over C_2_H_6_ because the incorporation of C_2_H_4_ binding sites such as unsaturated metal sites into MOF structures is much more easily achieved.^19–21^ However, these C_2_H_4_-selective MOFs are not ideal adsorbents in practical MTO separation as the pure C_2_H_4_ product must be obtained from the additional desorption process. Thus the development of C_2_H_6_-selective MOFs to produce the pure C_2_H_4_ product directly at the outlet is highly desired. For C_3_H_6_/C_2_H_4_ separation, previous reports demonstrated that the strategy of utilizing polar functional groups such as open metal sites (OMSs) in MOFs is feasible due to the higher acidity and more –CH groups in C_3_H_6_ in comparison to C_2_H_4_.^1,22^ However, this strategy is paradoxical to the C_2_H_6_-selective MOFs because the OMSs in MOFs prefer to interact strongly with C_2_H_4_ instead of C_2_H_6_, leading to preferential adsorption of C_2_H_4_ over C_2_H_6_, while C_2_H_4_ purification and C_3_H_6_ recovery are both important in order to produce high-purity C_2_H_4_ and C_3_H_6_ products in the MTO process. Therefore, the development of MOF materials for the efficient separation of both C_2_H_6_/C_2_H_4_ and C_3_H_6_/C_2_H_4_ is a significant challenge.

Recent reports have indicated that enhancing the non-polarity of the pore surface in MOFs can facilitate the selective adsorption of C_2_H_6_ over C_2_H_4_.^23–27^ This is because of the higher polarizability of C_2_H_6_ (44.7 × 10^−25^ cm^3^) compared to that of C_2_H_4_ (42.52 × 10^−25^ cm^3^).^23^ At the same time, nonpolar pores generated by aromatic rings can offer more and stronger C–H⋯π interactions, resulting in enhanced paraffin affinity and selective C_2_H_6_ adsorption.^28–30^ On the other hand, as aromatic moieties such as benzene rings possess delocalized π electrons, the pore surface decorated with benzene rings can provide stronger π⋯π interactions between the host and C_3_H_6_ with a conjugated π system in comparison to C_2_H_4_ molecules.^31^ For example, Chen *et al.* reported that a C_3_H_6_-selective MOF ZJNU-401 exhibited preferential C_3_H_6_ adsorption and good C_3_H_6_/C_2_H_4_ selectivity, which can be attributed to suitable pore size and benzene ring decorated pore walls.^27^ Therefore, we speculate that the MOF structure with a nonpolar pore surface decorated by benzene rings and without OMSs could realize the efficient separation of both C_3_H_6_/C_2_H_4_ and C_2_H_6_/C_2_H_4_ mixtures.

Additionally, the practical application of high-performance MOFs faces several limitations, primarily high costs, challenges in scalability, and potential performance degradation. Ligand design is crucial for achieving desired structures and properties, but it often leads to significantly increased costs for adsorbents. The raw materials for synthesizing organic ligands can cost tens of dollars per gram, and the production of MOF adsorbents typically requires long reaction times, derailing scalable manufacturing. For example, the C_2_H_6_-selective benchmark MOF ZNU-10 is synthesized from the reaction of Cu(NO_3_)_2_·3H_2_O and mixed linkers *p*-C_2_B_10_H_10_–(COOH)_2_ and DABCO in DMF/MeOH/H_2_O solution, with the price not less than $7 per gram.^32^ Recently, Hou's group developed a high performance MOF adsorbent, MAC-4, which combines isophthalic acid and 3,5-dimethyl-1,2,4-triazole, and synthesis can be scaled-up with a cost as low as 1.35 $ per gram.^24^ The scale-up synthesis of MAC-4 is not an environment-friendly protocol due to the use of DMF solvent, and a sustainable synthesis process can accelerate the commercialization of MOF materials, such as CALF-20,^33,34^ MIL-100(Fe),^35,36^*etc.* To advance the industrial application of MOF adsorbents, it is essential to explore options that involve simple, green synthesis processes and very low costs, ensuring high scalability and practicality.

Taking above into consideration, herein we present an efficient C_3_H_6_- and C_2_H_6_-selective MOF adsorbent, Zn-hba, enabling the one-step purification of C_2_H_4_ and efficient recovery of C_3_H_6_ for the separation of MTO products. Zn-hba is synthesized using the common cheap ligand 4-hydroxybenzoic acid (HBA) and the late transition metal zinc (Zn) through a simple and scaled-up synthesis process, achieving a high yield of 42% (based on Zn) (see the Synthetic section for the detailed procedure). The simple structure avoids the existence of OMSs and features dense, periodic quadrate channels decorated with nonpolar benzene rings, facilitating efficient separation of C_2_H_6_/C_2_H_4_/C_3_H_6_ mixtures without performance degradation after cycling measurements. Zn-hba exhibits preferential adsorption of C_2_H_6_ and C_3_H_6_ over C_2_H_4_ with a record-high C_3_H_6_ packing density (257.54 g L^−1^) at 1 kPa and extreme high C_2_H_6_ packing density (186.94 g L^−1^) at 10 kPa. The Ideal Adsorption Solution Theory (IAST) selectivity for C_2_H_6_/C_2_H_4_ and C_3_H_6_/C_2_H_4_ is calculated to be 2.41 and 11.73, respectively. Theoretical calculations reveal that the stronger binding affinity for C_3_H_6_ and C_2_H_6_ over C_2_H_4_ between the nonpolar pore surface and guest molecules is responsible for the C_3_H_6_- and C_2_H_6_-selective adsorption. Breakthrough experiments confirm the good separation performance of Zn-hba for C_2_H_6_/C_2_H_4_ and C_3_H_6_/C_2_H_4_ mixtures even under humid conditions. Moreover, Zn-hba could separate the ternary C_2_H_6_/C_3_H_6_/C_2_H_4_ mixtures to produce high-purity C_2_H_4_ (>99.9%) and C_3_H_6_ (>99.5%) with a productivity of 3.32 and 19.42 L kg^−1^, respectively. In addition, all reagents used for the synthesis of Zn-hba are commercially available and extremely low cost. Despite the fact that the synthesis is not very sustainable, Zn-hba exhibits a significant cost advantage. The cost per gram of Zn-hba is as low as $0.14, making it comparable to that of commercial zeolites and nearly one-tenth of the price of MAC-4.

## Results and discussion

### Synthesis and characterization

The evaporation of methanol in the *n*-amyl alcohol/MeOH solution containing Zn(OAc)_2_·2H_2_O and 4-hydroxy benzoic acid (hba) yielded colorless needle-like crystals of Zn-hba according to the literature.^37^ The scale-up synthesis of Zn-hba also has been investigated *via* using an alternative simple protocol. As shown in Fig. S1, *n*-amyl alcohol was added into the MeOH solution containing Zn(OAc)_2_·2H_2_O and 4-hydroxy benzoic acid under heating conditions at 100 °C in a round flask to obtain the final microcrystalline Zn-hba sample, and the solvent methanol was recovered during the reaction. These mild synthetic conditions and simple synthetic procedures can produce around 5 g of Zn-hba with a high yield of 42% in 12 hours through one single reaction. Moreover, compared to most porous MOFs applied to gas adsorptive separation, the raw materials for synthesis of Zn-hba are commercially available and the price is very low. The cost of the Zn-hba material is calculated to be $0.14 per kg (Table S1), which makes it comparable to that of commercial zeolites and nearly one-tenth of the price of MAC-4. It is noted that Zn-hba made with this scalable synthesis method exhibited good crystallinity and high phase purity, which was confirmed by powder X-ray diffraction (PXRD) measurements (Fig. S2).

**Fig. 1:**
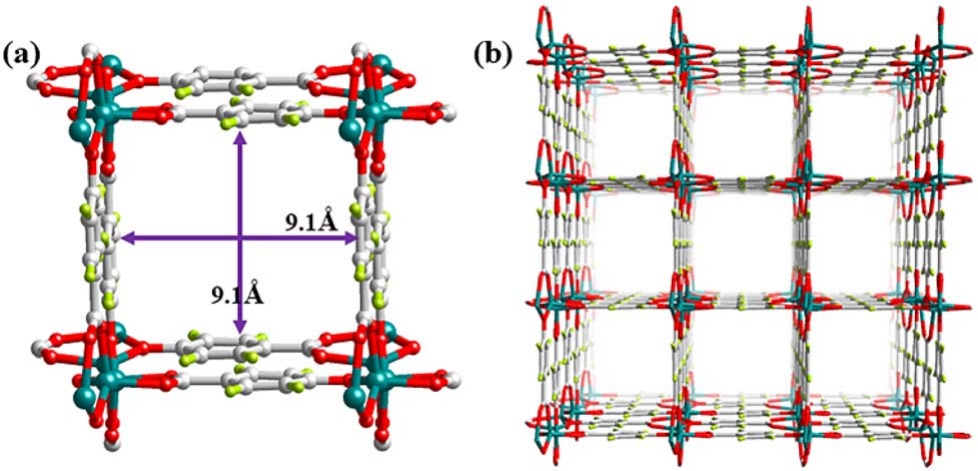
(a) Schematic illustration of a 1 D channel decorated with benzene rings and (b) the 3D network of Zn-hba.

The network of Zn-hba is constructed with 1D tetrahedral zinc(ii)-based helical chains as the second building unit (SBU) connected to μ_2_-phenolate and carboxylate groups. The hba linkers extend from the metal chains in four directions and perpendicular to the 1D helical chains. Furthermore, each hba linker is linked to two adjacent helical chains, resulting in a 3D structure of 9.1 Å × 9.1 Å square channels with a formula of Zn(hba)·*x*H_2_O (Fig. 1). Based on the Platon calculation, the framework of Zn-hba exhibits a porosity of 37.9%. Additionally, the 1D square channels in the 001 direction are decorated with aromatic moieties and the open metal sites are completely avoided in the structure, providing a nonpolar pore surface that facilitates selective C_3_H_6_ and C_2_H_6_ adsorption over C_2_H_4_ for Zn-hba. The PXRD pattern of as-synthesized samples is consistent with the corresponding simulated one from single crystal data, indicating that its bulk product was pure phase (Fig. S2). The thermogravimetric analysis (TGA) indicated that Zn-hba can be completely desolvated (Fig. S3), facilitating the examination of their permanent porosities. Nitrogen (N_2_) adsorption isotherms at 77 K showed type-I curves of Zn-hba (Fig. S4), with a saturated loading of 177 cm^3^ g^−1^, indicating a close Brunauer–Emmett–Teller (BET) surface area of 672 m^2^ g^−1^, and corresponding experimental pore volume of 0.259 cm^3^ g^−1^. The pore size distribution of 8 Å agrees with the calculated results from the crystal structure. Moreover, the chemical stability of Zn-hba has been examined. The variable temperature VT-PXRD measurements show that Zn-hba can retain its structural rigidity up to 500 °C (Fig. S5). This ultrahigh thermal stability is rare among MOFs and surpasses that of all Zn–MOFs^23,24,38–42^ to the best of our knowledge. Besides, Zn-hba also exhibits high stability in humid air for one week (Fig. S6). Overall, these data demonstrate that Zn-hba is a robust porous MOF for gas adsorption.

### Gas adsorption properties

**Fig. 2:**
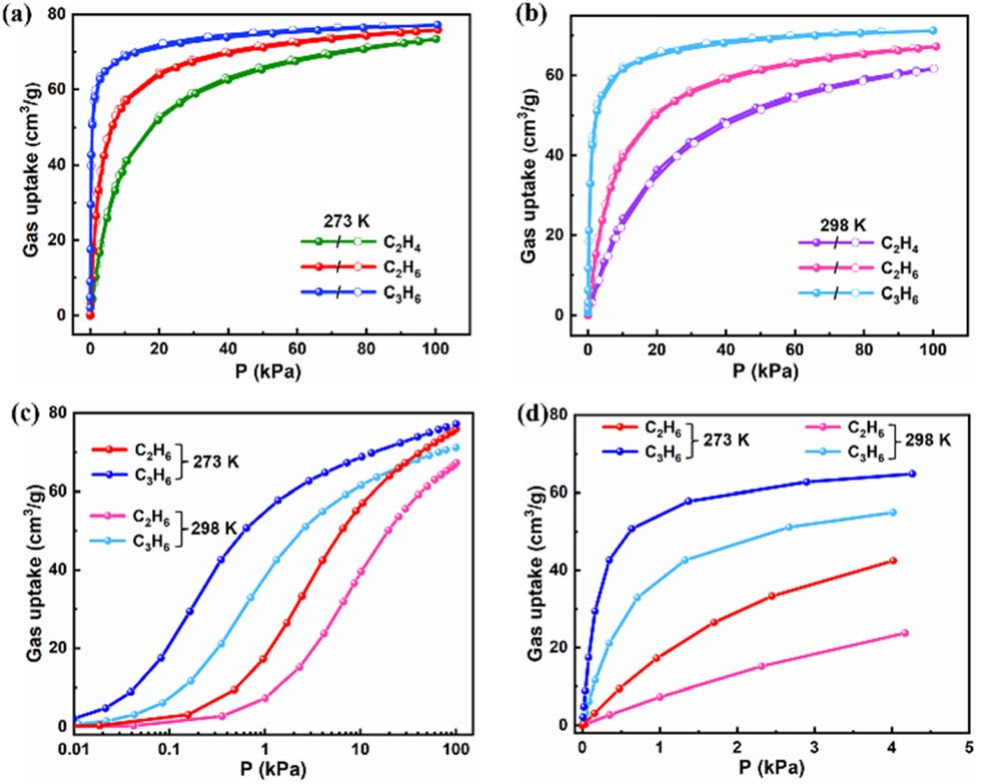
Gas adsorption isotherms of C_2_H_4_, C_2_H_6_, and C_3_H_6_ on small-scale Zn-hba at (a) 273 K and (b) 298 K; logarithmic-scale plots within the range of (c) 0–100 kPa and (d) 0–5 kPa at 273/298 K.

The well-developed channels combined with the nonpolar pore surface and high stability encouraged us to investigate the gas adsorption performance of Zn-hba for light hydrocarbons. Single-component gas adsorption isotherms for C_2_H_6_, C_2_H_4_ and C_3_H_6_ were collected on Zn-hba small-scale samples at 273/298 K under 100 kPa. As illustrated in Fig. 2a and b, the maximum uptake of 75.9/67.3 cm^3^ g^−1^ for C_2_H_6_ and 77.2/71.2 cm^3^ g^−1^ for C_3_H_6_ at 273/298 K, were both much higher than that of 73.4/61.7 cm^3^ g^−1^ for C_2_H_4_. Thus the adsorption capacity is in the order of C_3_H_6_ > C_2_H_6_ > C_2_H_4_, indicating a reversed adsorption behaviour in Zn-hba, which is quite rare among reported MOFs.^23–25,27,43^ Remarkably, Zn-hba exhibits abundant C_3_H_6_ and C_2_H_6_ adsorption at extremely low pressure at 273 or 298 K (Fig. 2c and d). The adsorption of C_3_H_6_ is record-high with an uptake of 38.91 cm^3^ g^−1^ at 1 kPa and 298 K, being the highest among all MOFs for C_3_H_6_-selective adsorption (Table S2). The adsorption capacity of C_2_H_6_, with a value of 39.54 cm^3^ g^−1^ at 10 kPa and 298 K, is also the highest among most MOFs for C_2_H_6_-selective adsorption, except slightly lower than that of NTU-70P (40.3 cm^3^ g^−1^)^40^ (Table S3). Afterwards, the packing density of C_3_H_6_ was calculated to be 258 g L^−1^, which is the highest one at 1 kPa when compared with that of reported MOFs (Table S2). The C_2_H_6_ packing density was estimated to be 187 g L^−1^, being the second highest value at 10 kPa in comparison to other MOFs (Table S3). It is worth noting that the C_2_H_6_ and C_3_H_6_ packing density outperforms that of some of the best-performing MOF materials such as Zn-BPZ-TATB,^23^ MAC-4 (ref. 24) and ZJUN-401.^27^ Meanwhile, the adsorption of C_2_H_4_, especially in the low-pressure regions, is quite low. Moreover, it is also clear that the slopes of C_2_H_6_ and C_3_H_6_ adsorption isotherms are much steeper than that of C_2_H_4_, indicating that the adsorption affinity in Zn-hba follows the order of C_3_H_6_ > C_2_H_6_ > C_2_H_4_, which suggests that Zn-hba has great potential for C_3_H_6_/C_2_H_6_/C_2_H_4_ gas mixture separation in the MTO process. Importantly, the scale-up synthesized Zn-hba exhibits similar adsorption isotherms to C_2_H_4_, C_2_H_6_ and C_3_H_6_ in comparison with the small-scale synthesized single crystal samples (Fig. S7). To further assess the durability of Zn-hba for gas adsorption performance, we conducted cyclic adsorption experiments. The continuous C_2_H_6_, C_2_H_4_ and C_3_H_6_ adsorption measurements without reactivation at 298 K showed the uptake of C_2_H_6_, C_2_H_4_ and C_3_H_6_ and no obvious decrease for atleast five adsorption cycles (Fig. S8), demonstrating excellent recyclability and regeneration capability of the material. The high crystallinity of Zn-hba post-experimentation was confirmed by PXRD analysis and N_2_ adsorption results (Fig. S9). No obvious change in PXRD peaks and N_2_ adsorption curves at 77 K (Fig. S9) was observed, indicating that Zn-hba possesses excellent durability and recyclability for this gas adsorption.

The adsorption enthalpies (*Q*_st_) were determined from sorption isotherms *via* the Clausius–Clapeyron equation at 298 K and 273 K to appraise the interaction strengths of the framework for C_2_H_4_, C_2_H_6_ and C_3_H_6_. The *Q*_st_ values at zero-coverage of C_3_H_6_, C_2_H_6_ and C_2_H_4_ were computed to be 31.0, 26.9 and 26.1 kJ mol^−1^ (Fig. S10), respectively, corroborating that C_3_H_6_ and C_2_H_6_ have stronger affinity with the framework than C_2_H_4_. It is noteworthy that the *Q*_st_ values of all three gases are relatively low due to the nonpolar pore surface of Zn-hba, indicating that the regeneration treatment is simple and consumes a low amount of energy. In fact, a simple activation treatment at 373 K for 30 minutes prior to each cycle was sufficient to regenerate fully reproducible adsorption profiles for C_3_H_6_, C_2_H_6_ and C_2_H_4_.

**Fig. 3:**
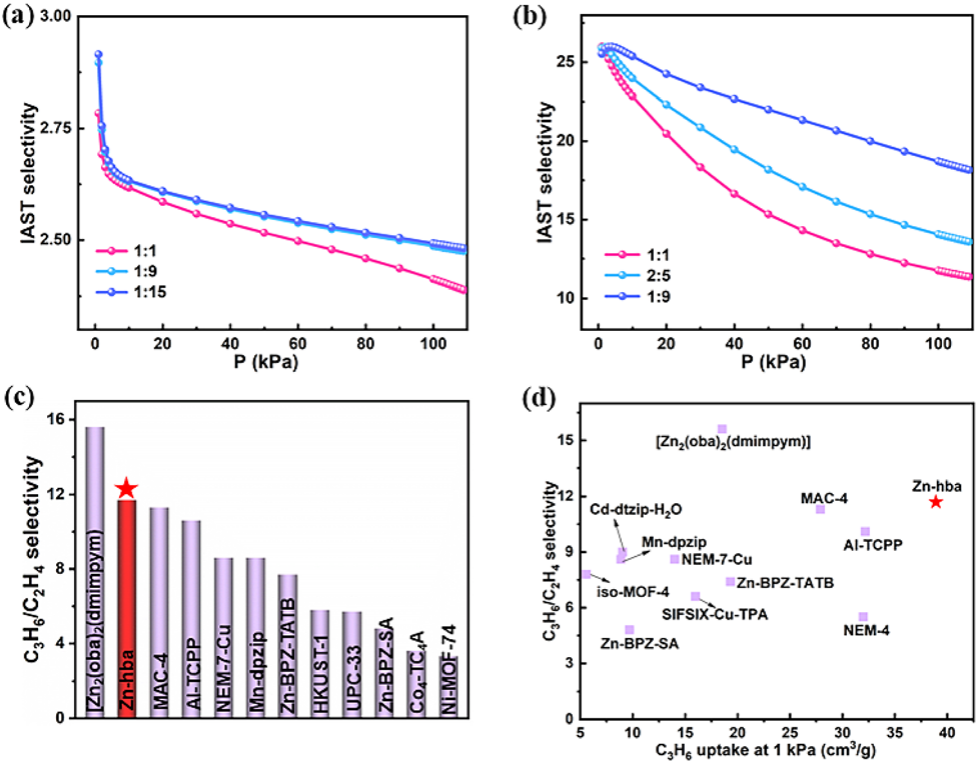
IAST selectivity curves of Zn-hba for (a) C_2_H_6_/C_2_H_4_ and (b) C_3_H_6_/C_2_H_4_ mixtures at 298 K; (c) comparisons of the selectivity in Zn-hba and some benchmark materials for C_3_H_6_/C_2_H_4_ (1/1); (d) comparison of C_3_H_6_ uptake at 1 kPa and C_3_H_6_/C_2_H_4_ IAST selectivity for Zn-hba and other benchmark materials.

As discussed above, Zn-hba exhibits a higher adsorption capacity of C_3_H_6_ and C_2_H_6_ than that of C_2_H_4_, making it a potential material for C_2_H_4_ purification in MTO application. Thus we have examined the separation of binary gas mixtures in the range of 0–100 kPa using ideal adsorption solution theory (IAST). As shown in Fig. 3a and S11, Zn-hba exhibits high selectivity values of 2.41/2.49/2.50, and 2.61/2.70/2.7 for 1/1, 1/9, and 1/15 C_2_H_6_/C_2_H_4_ mixtures at 298/273 K and 100 kPa, respectively.

The selectivity for the equimolar C_2_H_6_/C_2_H_4_ mixture at 298 K and 100 kPa is comparable to that of many top-performing C_2_H_6_-selective MOFs such as ZIF-8,^44^ PCN-250,^45^ and MAC-4 (ref. 24) (Fig. S12). For C_3_H_6_/C_2_H_4_ mixtures, the selectivity values were estimated to be 11.73/14.05/18.70, and 16.33/18.80/22.93 for 1/1, 2/5, and 1/9 C_3_H_6_/C_2_H_4_ mixtures at 298/273 K and 100 kPa, respectively (Fig. 3b and S13). Notably, this selectivity for the equimolar C_3_H_6_/C_2_H_4_ mixture at 298 K and 100 kPa is the highest among reported C_3_H_6_-selective MOFs,^22,23,25,46^ but only lower than that of Zn_2_(oba)_2_(dmipym) (15.6)^26^ (Fig. 3c and d).

**Fig. 4:**
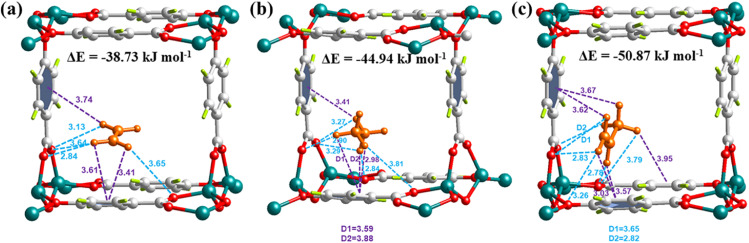
The DFT-calculated configurations of (a) C_2_H_4_, (b) C_2_H_6_, and (c) C_3_H_6_ in Zn-hba. Green, Zn; red, O; gray, C; light green, H; orange, adsorbed gas molecules.

To in-depth understand the adsorption mechanism of C_3_H_6_, C_2_H_6_ and C_2_H_4_ in Zn-hba, theoretical calculations based on first-principles dispersion-corrected density functional theory (DFT-D) were performed to reveal the host–guest interactions between the framework and adsorbates. As illustrated in Fig. 4 and S14, the preferential adsorption sites of C_3_H_6_, C_2_H_6_ and C_2_H_4_ are similar and located within the square channels. In Zn-hba, one C_2_H_4_ molecule interacts with two carboxylate groups in hba linkers *via* four C–H⋯O interactions (2.84, 3.13, 3.64 and 3.65 Å) and two aromatic moieties *via* three C–H⋯π interactions (3.41, 3.61 and 3.74 Å), providing a static binding energy of −38.73 kJ mol^−1^ (Fig. 4a). For C_2_H_6_, there are five C–H⋯O interactions (2.84, 2.90, 3.27, 3.29 and 3.81 Å) between the gas molecule and carboxylates, and four C–H⋯π interactions (2.98, 3.41, 3.59 and 3.88 Å). The average binding energy for these C_2_H_6_ adsorption sites is calculated to be −44.94 kJ mol^−1^, indicating a stronger binding affinity between the alkane adsorbate and nonpolar pore surface with aromatic rings (Fig. 4b). Due to more hydrogen atoms and the larger size of the C_3_H_6_ molecule, there are more contacts involving C–H⋯O interactions (2.78, 2.82, 2.83, 3.26, 3.65 and 3.79 Å) between C_3_H_6_ and carboxylate/hydroxyl groups in hba and C–H⋯π interactions (3.03, 3.57, 3.62, 3.67 and 3.95 Å) between C_3_H_6_ and benzene rings, with a much stronger binding energy of −50.87 kJ mol^−1^, resulting in an enhanced host–guest interaction to facilitate the capture of propylene in the Zn-hba framework (Fig. 4c). These DFT calculation results provide a rational explanation for the experimental observations of preferential adsorption of C_2_H_6_ and C_3_H_6_ over C_2_H_4_. Apart from the thermodynamics of host–guest interactions, the kinetic adsorption effect is also very important for assessing porous materials' performance. Therefore, the adsorption kinetics were also studied using molecular dynamics (MD) simulations.

As shown in Fig. S15, the diffusion coefficients (*D*_s_) of the three gas molecules were estimated to be 1.73 × 10^−8^ m^2^ s^−1^ for C_2_H_4_, 1.27 × 10^−8^ m^2^ s^−1^ for C_2_H_6_ and 1.05 × 10^−8^ m^2^ s^−1^ for C_3_H_6_, respectively, demonstrating faster diffusion behavior of C_2_H_4_ and C_2_H_6_ over C_3_H_6_. Additionally, kinetic mechanisms were investigated *via* adsorption experiments (Fig. S16). The adsorption rates were collected from the adsorption analyzer and the experimental kinetic diffusion coefficients (*K*_s_) were determined to be 4.3 × 10^−2^, 2.5 × 10^−2^ and 1.1 × 10^−2^ for C_2_H_4_, C_2_H_6_ and C_3_H_6_, respectively. The larger *K*_s_ of C_2_H_6_ and C_3_H_6_ than C_2_H_4_ indicates the faster adsorption kinetics of C_2_H_6_ and C_3_H_6_ compared to that of C_2_H_4_ in Zn-hba. The experimental data are consistent with the MD simulation results, demonstrating that smaller adsorbate molecular size causes faster guest diffusion. In brief, we thus reason that the preferential adsorption of C_2_H_6_ and C_3_H_6_ over C_2_H_4_ is determined by thermodynamic mechanisms instead of kinetic mechanisms.

### Breakthrough experiments

**Fig. 5:**
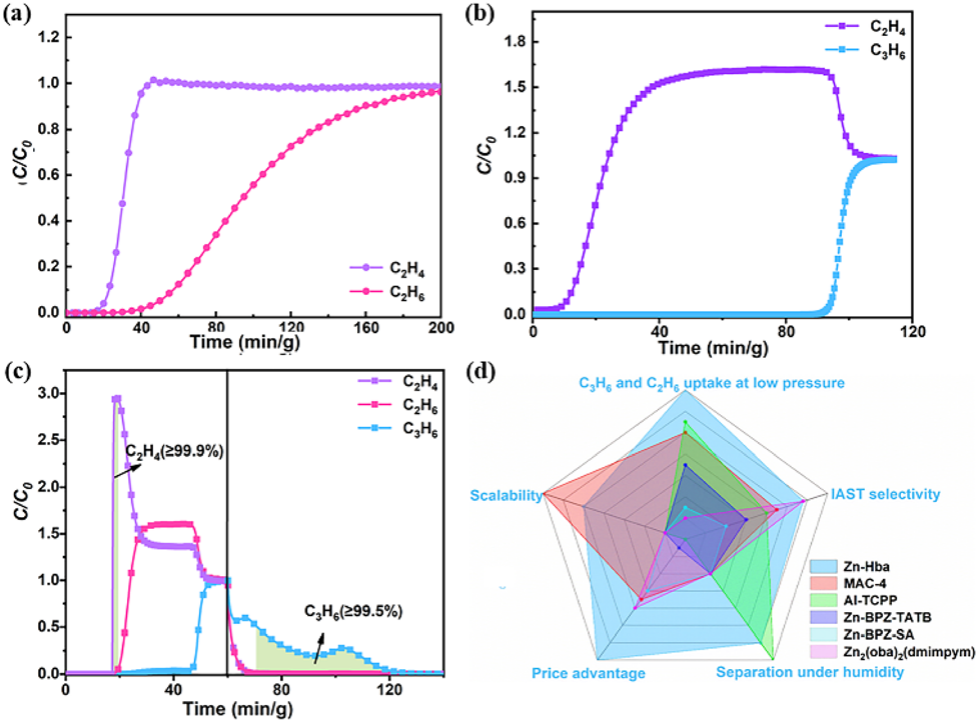
(a) Breakthrough curves for C_2_H_6_/C_2_H_4_ (v/v, 1/15) mixtures at 298 K; (b) breakthrough curves for C_3_H_6_/C_2_H_4_ (v/v, 5/5) mixtures at 298 K; (c) breakthrough curves for the C_2_H_6_/C_3_H_6_/C_2_H_4_ mixture (v/v/v, 33.3/33.3/33.3) at 298 K; (d) comprehensive comparisons for Zn-hba and other porous materials.

To evaluate the practical C_2_H_6_/C_2_H_4_ separation performance of Zn-hba, dynamic breakthrough experiments were performed on Zn-hba large-scale samples for C_2_H_6_/C_2_H_4_ (v/v, 5/5, 1/9, and 1/15) mixtures using Ar as the carrier gas with a total flow rate of 10 mL min^−1^ at 298 K. C_2_H_4_ eluted first from the column to produce the high-purity C_2_H_4_ (>99.9%) product (Fig. 5a and S17). The separation factor was estimated to be 2.36, 2.75 and 2.98 for 5/5, 1/9, and 1/15 C_2_H_6_/C_2_H_4_ mixtures, respectively, which are similar to the IAST selectivities. The separation performance of the Zn-hba material for C_3_H_6_/C_2_H_4_ (v/v: 5/5, 2/5, and 1/9) mixtures was also verified under similar conditions. As shown in Fig. 5b and S18, C_2_H_4_ passed through the column rapidly while C_3_H_6_ flowed out slowly at 92/163/172 min g^−1^, respectively. Notably, these breakthrough time intervals for C_3_H_6_ in Zn-hba are the longest among most MOFs for C_3_H_6_/C_2_H_4_ separation, such as Zn-BPZ-TATB,^23^ MAC-4 (ref. 24) and ZJNU-401,^27^ suggesting the excellent separation ability of Zn-hba for C_3_H_6_/C_2_H_4_. Subsequently, the polymer-grade C_2_H_4_ (>99.95%) was estimated to be 56.49/85.17/120.00 L kg^−1^, superior to those of [Zn_2_(oba)_2_(dmimpym)],^26^ Zn-BPZ-SA^47^ under similar conditions. As pure C_3_H_6_ is another very important product in the MTO process, high-purity C_3_H_6_ (>99.94%) can be obtained by the desorption experiments *via* Ar purging. As a result, the productivity of C_3_H_6_ for the C_3_H_6_/C_2_H_4_ mixture (v/v, 5/5) was calculated to be 25.81 L kg^−1^, which is comparable to that of Cd-dtzip-H_2_O (Fig. S19).^48^ Furthermore, Zn-hba also exhibited good C_3_H_6_/C_2_H_4_ separation performance under humid conditions. The breakthrough time was almost the same for 3 cycles of breakthrough experiments under 50% and 100% humid conditions (Fig. S20 and S21), indicating the excellent moisture resistance of Zn-hba for C_3_H_6_/C_2_H_4_ separation. The structural stability of Zn-hba was confirmed by PXRD and N_2_ adsorption results (Fig. S22 and S23), proving its excellent humidity-resistant recyclability and durability. Additionally, as illustrated in Fig. S24 and S25, Zn-hba shows good recycling performance for the separation of equimolar C_2_H_6_/C_2_H_4_ and C_3_H_6_/C_2_H_4_ mixtures at 298 K, making it a potential material for industrial C_2_H_4_ purification.

During the real industrial process, there are some light hydrocarbons in the MTO products, which is a significant challenge for high-purity C_3_H_6_ recovery and C_2_H_4_ purification. Therefore, the dynamic breakthrough measurements for ternary C_2_H_6_/C_3_H_6_/C_2_H_4_ mixtures with various ratios were performed at 298 K to evaluate the separation performance of Zn-hba. As shown in Fig. 5c, for the 33.3/33.3/33.3 ternary mixtures, C_2_H_4_ passed through the column rapidly to produce high-purity C_2_H_4_ (>99.9%) with a productivity of 3.32 L kg^−1^, following by C_2_H_6_ detected from the outlet, while C_3_H_6_ occurred until around 46 min. Then the desorption experiments were performed by purging with He gas with a flow rate of 5 mL min^−1^ at 373 K. As a result, 19.42 L kg^−1^ C_3_H_6_ (>99.5%) were recovered from the ternary mixtures in one single adsorption/desorption process. Moreover, when the ratios of ternary mixtures were set up to be 1/9/90 and 2/10/25, Zn-hba still exhibited efficient separation performance (Fig. S26 and S27), making it an excellent adsorbent for C_2_H_4_ purification and C_3_H_6_ recovery in MTO product separation. Overall, Zn-hba obtains good scores in uptake at low pressure, IAST selectivity, scalability, price and separation under humidity, which highlights its high promise for real industrial separation (Fig. 5d).

## Conclusions

In summary, we developed a scalable and simple synthetic method to produce a low-cost and robust MOF, Zn-hba. Its production cost is only $0.14 per kg, significantly lower than that of most reported MOF materials. Moreover, the low polarity pore surface promotes that Zn-hba prefers adsorbing C_3_H_6_ and C_2_H_6_ over C_2_H_4_ and yields a record-high propylene packing density and exceptionally high ethane packing density at low pressure, which leads to significant C_3_H_6_/C_2_H_4_ and C_2_H_6_/C_2_H_4_ selectivities, exceeding that of most C_3_H_6_ and C_2_H_6_-selective MOFs. DFT calculations reveal that there are more multiple supramolecular interactions and a stronger binding affinity between the nonpolar pore surface of Zn-hba and C_3_H_6_ and C_2_H_6_ in comparison to C_2_H_4_. Breakthrough experiments confirm the good separation performance of Zn-hba for C_2_H_6_/C_2_H_4_ and C_3_H_6_/C_2_H_4_ mixtures with the longest breakthrough time intervals. More importantly, Zn-hba maintains its C_3_H_6_/C_2_H_4_ separation ability after multiple cycles of breakthrough experiments at high humidity. In addition, Zn-hba can produce polymer-grade C_2_H_4_ (>99.9%) and high-purity C_3_H_6_ (>99.5%) from ternary C_3_H_6_/C_2_H_6_/C_2_H_4_ mixtures *via* one adsorption–desorption process. In general, the synthetic feasibility, low-cost, and easy scalability in combination with good separation performance make Zn-hba the most promising material for industrial gas separation. This work highlights the significance of a nonpolar pore environment in MOF materials to facilitate the challenging separation and practical industrial application.

## Author contributions

L. G. and H.-J. Y. conceived the idea. Y.-Y. Z. carried out the experiments and analyzed the data. L. G. and C.-Z. X. wrote the manuscript. All authors contributed to the preparation of the manuscript.

## Conflicts of interest

There are no conflicts to declare.

## Supplementary Material

SC-OLF-D5SC03946K-s001

## Data Availability

The data supporting this article have been included as part of the SI. Supplementary information is available: Synthetic procedure, experimental details, all characterisation data, additional adsorption and separation data. See DOI: https://doi.org/10.1039/d5sc03946k.
