# Supplementary material for: Scalable synthesis of a low-cost Zn–MOF with a nonpolar pore surface for efficient separation of methanol-to-olefin products
Source: Chem Sci. 2025 Sep 16;16(41):19381–8. doi: 10.1039/d5sc03946k (PMC12453045; doi:10.1039/d5sc03946k)
Supplement: SC-016-D5SC03946K-s001 [file SC-016-D5SC03946K-s001.pdf]

Supplementary Information (SI) for Chemical Science.
This journal is © The Royal Society of Chemistry 2025

Supporting Information

Scalable Synthesis of a Low-Cost Zn-MOF with Nonpolar Pore Surface for Efficient Separation of Methanol-to-Olefin Products

Yingying Zhang, [a] *Suyun Deng,* [a] *Xingye Cui,* [a] *Jixiang Yue,* [a] *Hongliang Huang,* [c] *Chaozhuang Xue,*[*b] *Huajun Yang,*[*a] *and Lei Gan*[*a]

[a] Jiangsu Key Laboratory of Biomedical Materials, School of Chemistry and Materials Science, Nanjing Normal University, Nanjing 210023, China
E-mail: huajunyang@nnu.edu.cn; leigan@nnu.edu.cn

[b] School of Chemistry and Chemical Engineering, Liaocheng University, Liaocheng, 252059, China
E-mail: czxuelc@163.com

[c] State Key Laboratory of Separation Membranes and Membrane Processes, School of Chemistry and Chemical Engineering, Tiangong University, Tianjin 300387, China

Materials

$Zn(CH_3COO)_2·2H_2O$, 4-hydroxy benzoic acid (HBA), *n*-Amyl alcohol, methanol (MEOH), dichloromethane (CH_2Cl_2) all were purchased commercially.

Synthesis of Zn-hba (small-scale synthesis)

Zn-hba was synthesized according to a previously published report with some modifications.[1] *n*-Amyl alcohol (20 mL) was added to a methanolic solution (15 mL) containing 4-hydroxy benzoic acid (0.0622 g, 0.451 mmol) and $Zn(CH_3COO)_2·2H_2O$ (0.100 g, 0.459 mmol). The reaction mixture is heated to 110 °C in an open glass vial. With continued heating the more volatile methanol was selectively lost from the reaction mixture. The removal of nearly all of the MeOH allowed the separation of colourless needle-like crystals. The phase purity was identified by the powder X-ray diffraction.

Scale-up synthesis of Zn-hba (large-scale synthesis)

n-Amyl alcohol (180 mL) was added to a methanolic solution (130 mL) containing 4-hydroxy benzoic acid (2.79 g, 20.23 mmol) and $Zn(CH_3COO)_2·2H_2O$ (4.5 g, 20.66 mmol). The reaction mixture is heated to 100 °C in an open flask. The white microcrystalline of Zn-hba was collected through filtration, and then dried in vacuum oven (Yield: 5.1 g, 42% based on Zn^{2+} ions). Prior to the PXRD measurements, we made a careful washing treatment for the synthesized samples: after filtration, the samples were purified with DMF and then soaked in DMF for 24 h, then the samples were washed with methanol for 3 times and soaked in dichloromethane for three days, and the solution was refreshed during each day, finally the samples were dried in vacuum oven. Then the phase purity was identified by the powder X-ray diffraction.

Measurements

Structure characterizations

Powder X-ray Diffraction (PXRD) measurements on as-synthesized Zn-hba by using a Rigaku SmartLab X-ray powder diffractometer with Cu-Kα radiation. Thermogravimetric (TGA) analysis was carried out on a HENVEN ZH1450 analyzer heated from ambient temperature to 800 ^{o}C under a nitrogen gas atmosphere with a heating rate of 10°C/min. Around 10 mg of samples were heated from room temperature to different temperatures with a heating rate of 10°C/min under nitrogen flow using HENVEN ZH1450 instrument. The samples were kept at targeted temperature for 0.5 hour and then were allowed to be cooled to room temperature naturally. The processed samples were then subject to PXRD experiments.

Gas Sorption Measurement

N_2, C_2H_4, C_2H_6 and C_3H_6 sorption measurements were carried out on Automatic High Performance Surface Area and Aperture Analyzer (BSD-660 A3M). Prior to the measurement, the as-synthesized small-scale sample was purified with DMF and then soaked in DMF for 24 h. Then the samples were washed with methanol for 3 times and soaked in dichloromethane for three days. During each day, the solution was refreshed. After solvent exchange, the upper solvent was decanted. The sample was first dried under N_2 flow gently and was subsequently transferred into the test tube. The degas program was set as heating to 250°C, then keeping 250 ^{o}C for 12 h in situ degassing.

Adsorption Kinetics

C_2H_4, C_2H_6 and C_3H_6 adsorption rates were carried out on BEL gas and vapor adsorption instrument.

Adsorption rate considering diffusion in pores is expressed by Equation (1).

$$\frac{d_{q_t}}{dt} = k_s a_p (q_A - q_{*B}) \quad (1)$$

With Henry (linear) adsorption equilibrium equation, relationship between amount of adsorption and pressure can be expressed by Equation (2):

$$q^* = H \times P \quad (2)$$

H: Equilibrium constant [m^3 / g], *p*: Pressure [Pa]

Thus, equation (3) for batch adsorbing operation is used.

$$W(q - q_0) = V(p_0 - p) \quad (3)$$

W: Mass of adsorbent [g], V: Fluid volume [m^3]

Equation (4) is derived from Equations (1) to (3).

$$q = \left(\frac{1}{\alpha + 1}\right) - \left(\frac{\alpha}{\alpha + 1}\right) exp\left[- \left(\frac{\alpha + 1}{\alpha}\right) k_s a_p t\right] \quad (4)$$

$$\alpha = \frac{v}{WH}\text{:}Separationfactor,t\text{:}Time[s]$$

As separation factor α becomes smaller, it indicates that adsorbate shifts from fluid phase to solid phase. By solving Equation (5) for p/p_0, the following equation is obtained:

$$\frac{P}{p_0} = 1 - \left(\frac{p_0}{p_0 - p_{en-1}}\right)\left(\frac{1}{\alpha + 1}\right)\left[1 - exp\left(- \left(\frac{\alpha + 1}{\alpha}\right) k_s a_p t\right)\right] \quad (5)$$

Equation (6) is obtained by solving the equation for relationship between amount of adsorption and pressure before and after adsorption measurement at the n-th point.

$$\frac{P - P_{en}}{P_0 - P_{en}} = 1 - \left(\frac{P_0}{P_0 - P_{en-1}}\right)\left(\frac{1}{\alpha + 1}\right)\left[1 - exp\left(- \left(\frac{\alpha + 1}{\alpha}\right) k_s a_p t\right)\right] \quad (6)$$

With the ideal gas state Equation (P = cRT), Equation (6) is transformed to the following equation, from which solution of analysis considering LDF approximation can be obtained.

$$\frac{c - c_{en}}{c_0 - c_{en}} = 1 - \left(\frac{c_0 - c_{en}}{c_0 - c_{en}}\right)\left(\frac{1}{\alpha + 1}\right)\left[1 - exp\left(- \left(\frac{\alpha + 1}{\alpha}\right) k_s a_p t\right)\right] \quad (7)$$

Wherein, relationship between each pressure value and density C is expressed by Equations (8) to (10), respectively

$$C_0 = \frac{P_V sinV_s + P_V d_{en-1} V_d}{V_s + V_d} \quad (8)$$

$$C = P_V st \quad (9)$$

$$C_{en} = P_V d_{en} \quad (10)$$

Isosteric Analysis of the Heat of Adsorption

The isosteric heats of adsorption for all the gases were calculated using the isotherms at 273 K and 298 K, following the Clausius-Clapeyron equation. It was done with the calculation program embedded in the software of Automatic High Performance Surface Area and Aperture Analyzer (BSD-660 A3M). High accuracy of the Q_{st} was found in all the calculations as evidenced by the linearity in the isosters.

Calculations of Ideal Adsorbed Solution Theory (IAST)

To evaluate the C_2H_4/C_2H_6 and C_2H_4/C_3H_6 separation performance, the selectivity was calculated by ideal adsorbed solution theory (IAST). Dual-Site Langmuir-Freundlich (DSLF) model was employed to fit the gas adsorption isotherms over the entire pressure range. DSLF model can be written as:

$$N = \frac{A_1 B_1 P^{1/n_1}}{1 + B_1 P^{1/n_1}} + \frac{A_2 B_2 P^{1/n_2}}{1 + B_2 P^{1/n_2}} \qquad (1)$$

Where N is the quantity adsorbed, p is the pressure of bulk gas at equilibrium with adsorbed phase, A_i is the saturation loadings for adsorption site i (i=1 or 2), and B_i are the affinity parameters. $1/n_i$ is the index of heterogeneity. The R factors for all the fitting are close to or higher than 99.999%.

The detailed methodology for calculating the amount of A and B adsorption from a mixture by IAST is described elsewhere. The adsorption selectivity is finally defined as:

$$selectivity = \frac{q_A / q_B}{p_A / p_B} \qquad (2)$$

where q_i (i = A or B) is the uptake quantity in the mixture and p_i is the feeding partial pressure of component i.

Density Functional Theory (DFT) Calculation

The spin-polarized density functional theory (DFT) calculations[2, 3] were carried out in the CP2K code.[4] All calculations employed a mixed Gaussian and planewave basis sets. Core electrons were represented with norm-conserving Goedecker-Teter-Hutter pseudopotentials,[5, 6] and the valence electron wavefunction was expanded in a double-zeta basis set with polarization functions along with an auxiliary plane wave basis set with an energy cut off of 450 eV. The generalized gradient approximation exchange-correlation functional of Perdew, Burke, and Enzerhof (PBE)[7] was used. Periodic structure of MOF was used in the DFT calculation and each configuration was optimized with the Broyden-Fletcher-Goldfarb-Shanno (BGFS) algorithm with SCF convergence criteria of 1.0×10^{-6} au. The van der Waals correction of Grimme's DFT-D3 model was also adopted.

The adsorption energy between the benzene molecule and the MOFs was calculated using the following equation:

$$\Delta E = E_{GAS@MOF} - E_{MOF} - E_{GAS} \quad (S1)$$

In Eq. (S1), $E_{GAS@MOF}$ and E_{MOF} represent the total energies of the MOF with and without the adsorption of benzene molecule, respectively. E_{GAS} is the total energy of the benzene molecule. According to this equation, a negative adsorption energy corresponds to a stable adsorption structure.

Molecular dynamics (MD) simulations

The classical molecular dynamics (MD) simulations were performed on the molecular loading results obtained by GCMC simulations. The initial configurations for the MD simulations were produced by the GCMC simulation. The framework and the gas molecule were both deemed as rigid character. The constant-volume & temperature (NVT) ensemble was used to simulate the dynamic processes. The electrostatic interactions and the van der Waals interactions were evaluated by the Ewald summation method, with a Buffer width of 0.5 Å. The diffusion coefficients (Ds) of gas molecules were obtained through fitting the slope of simulated mean square displacement (MSD)[8].

Breakthrough measurements

The breakthrough experiments were performed on the Multi-component Adsorption Breakthrough Curve Analyzer (BSD-MAB) at 298K and 100 kPa. The activated large-scale Zn-hba sample (about 0.3 g) was filled into a packed column of ϕ 4 mm × 40 mm, and then the packed column was washed with He at a rate of 10 mL/min at 523 K for 720 minutes to further activate the samples. Between two breakthrough experiments, the adsorbent was regenerated by He flow of 10 mL/min for 120 min at 373 K to guarantee a complete removal of the adsorbed gas.

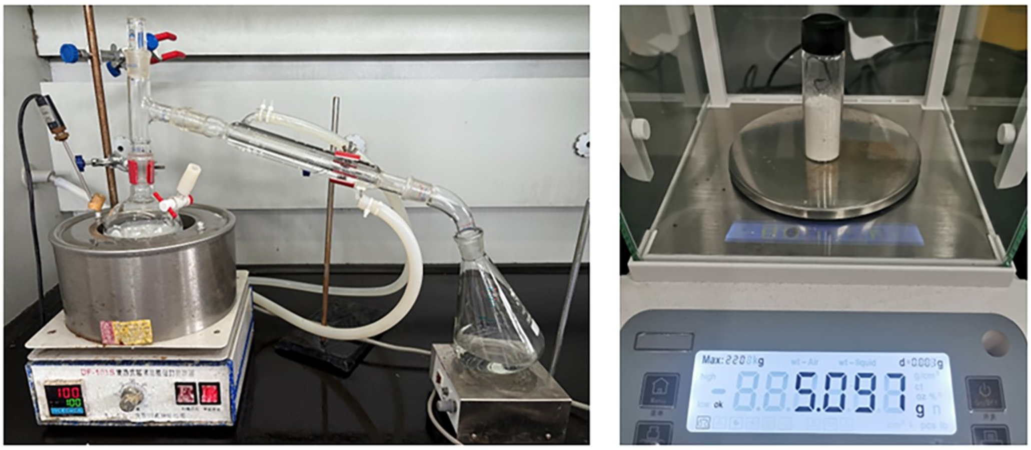

Fig. S1 Photographs of the scale-up synthesis of Zn-hba.

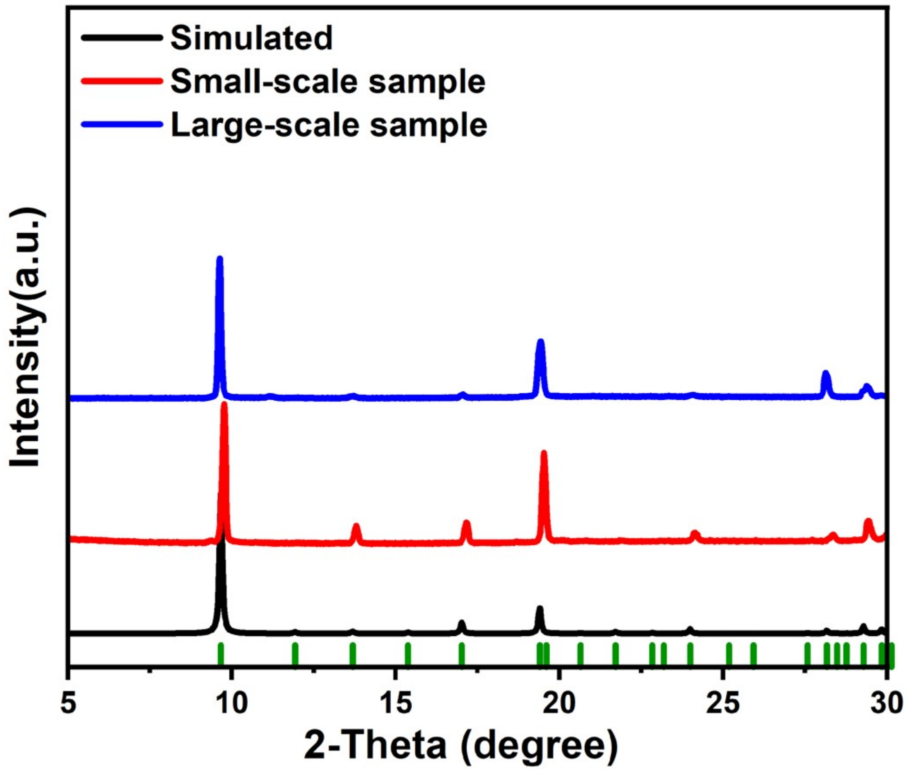

Fig. S2 PXRD pattern comparisons for Zn-hba samples.

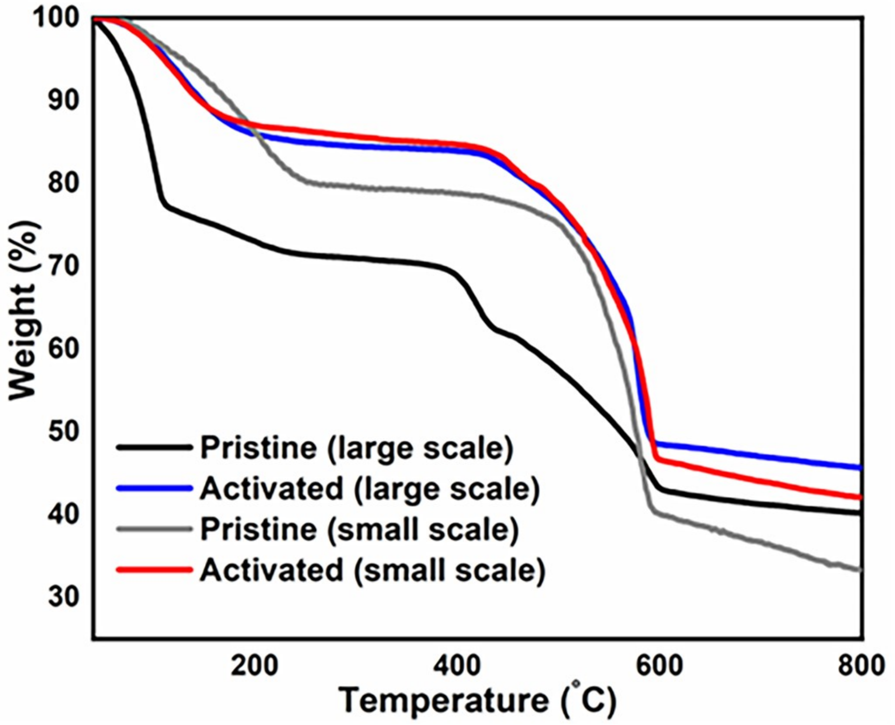

Fig. S3 TGA comparisons for synthesized and activated Zn-hba.(The results indicate that the TGA property of large-scale sample remains unchanged compared to small-scale sample after activation.)

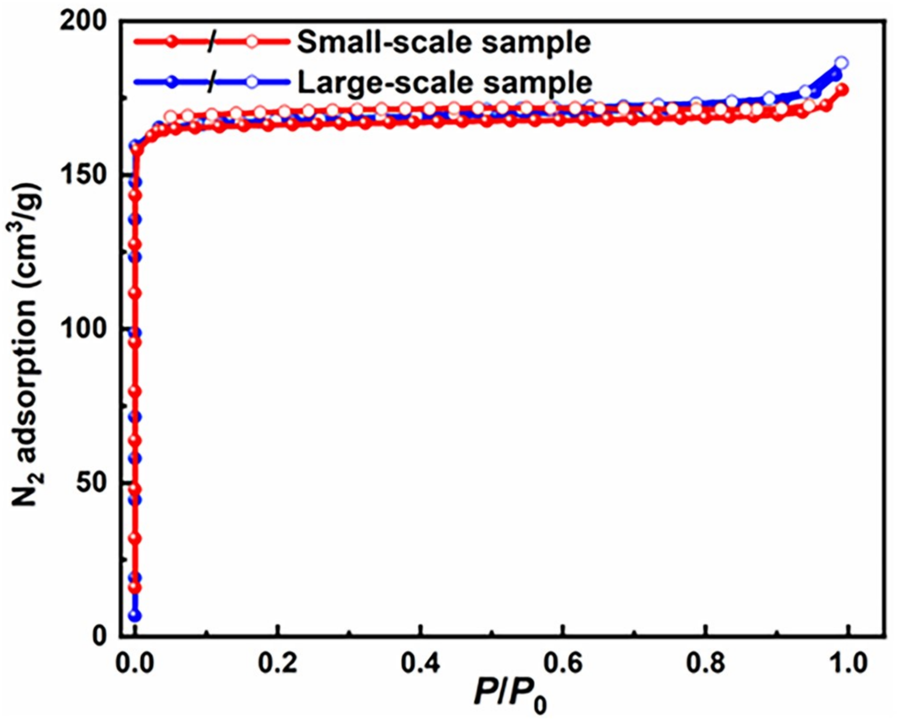

Fig. S4 N_2 adsorption isotherms at 77 K for Zn-hba. (The results indicate that the porosity of large-scale sample remains unchanged compared to small-scale sample after activation.)

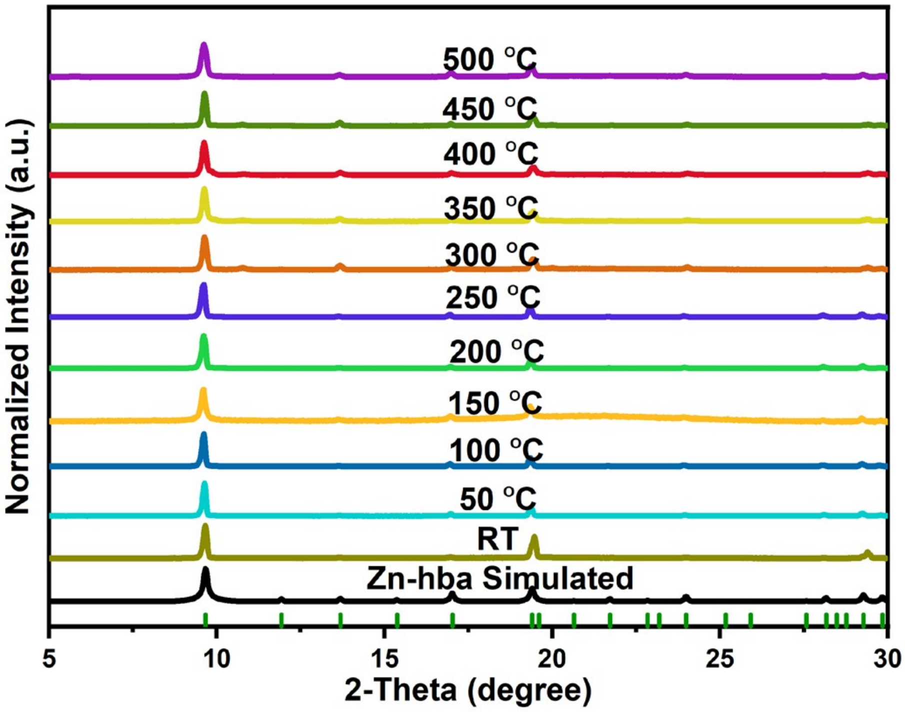

Fig. S5 Variable-Temperature PXRD patterns of the small-scale synthesized sample.

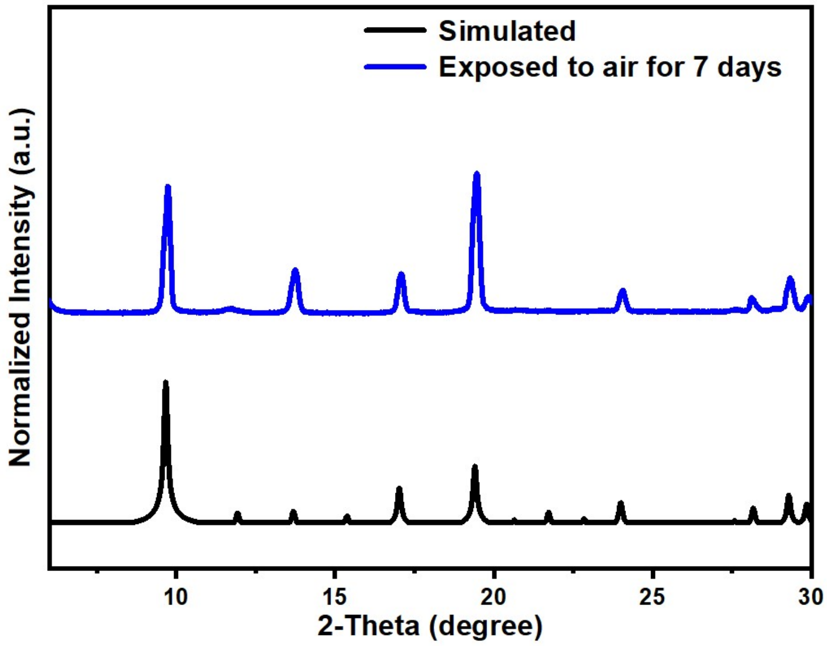

Fig. S6 PXRD patterns of Zn-hba after exposed to air for 7days.

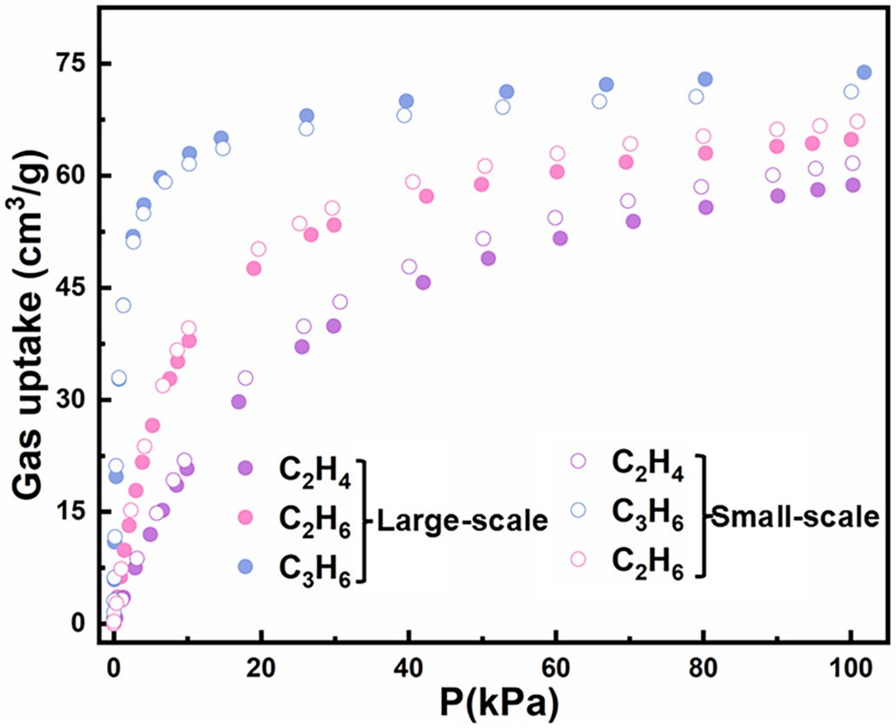

Fig. S7 Adsorption isotherm comparisons of C_2H_4, C_2H_6, and C_3H_6 at 298 K for small-scale Zn-hba single-crystal samples and scale-up synthesized Zn-hba powder samples.

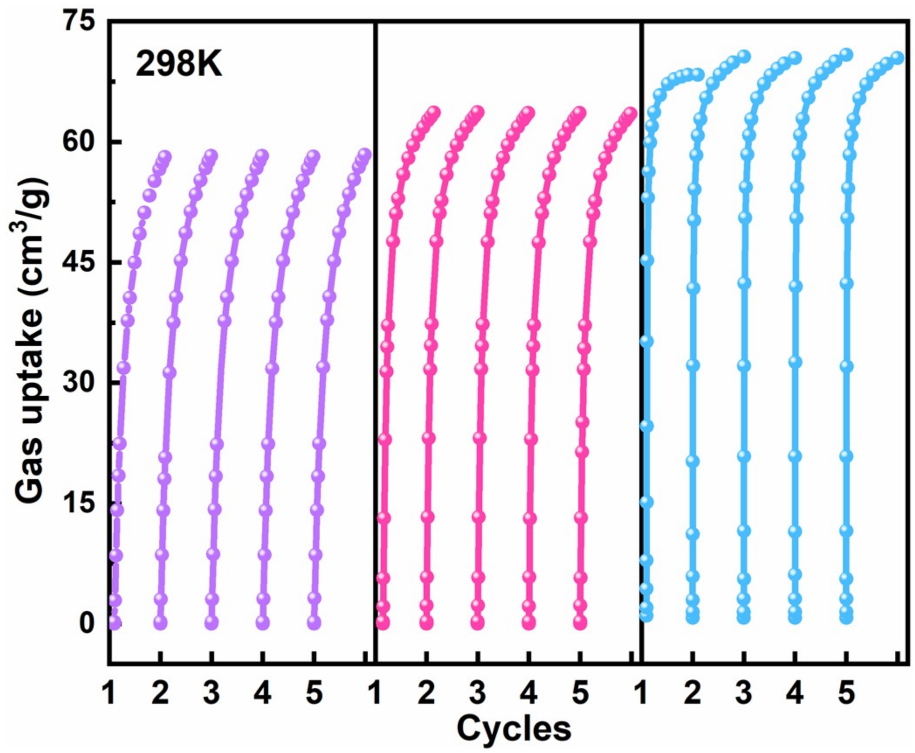

Fig. S8 Cycling C_2H_4 (purple), C_2H_6 (pink) and C_3H_6 (blue) adsorption measurements at 298 K for small-scale synthesized samples.

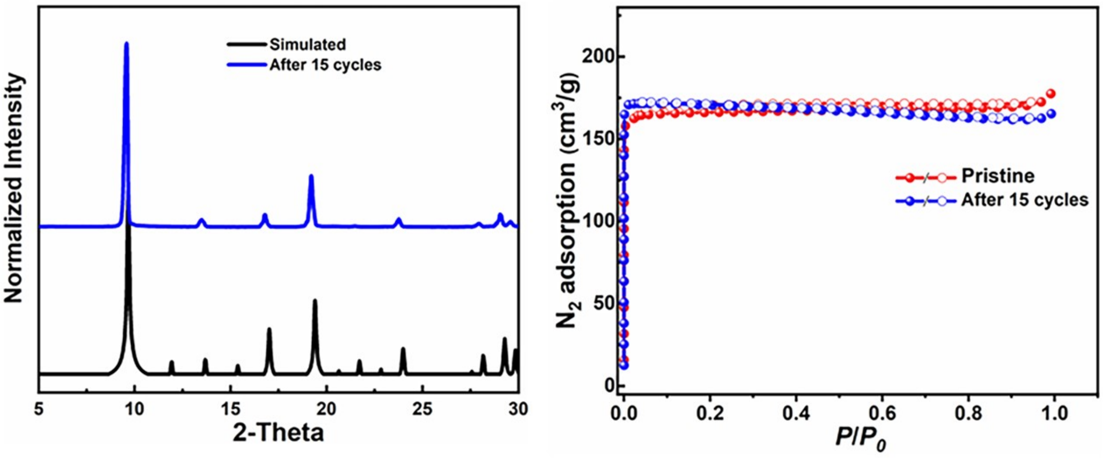

Fig. S9 The comparisons of PXRD and N_2 adsorption isotherms at 77 K for pristine small-scale synthesized sample and the sample after 15 cycles of gas adsorption.

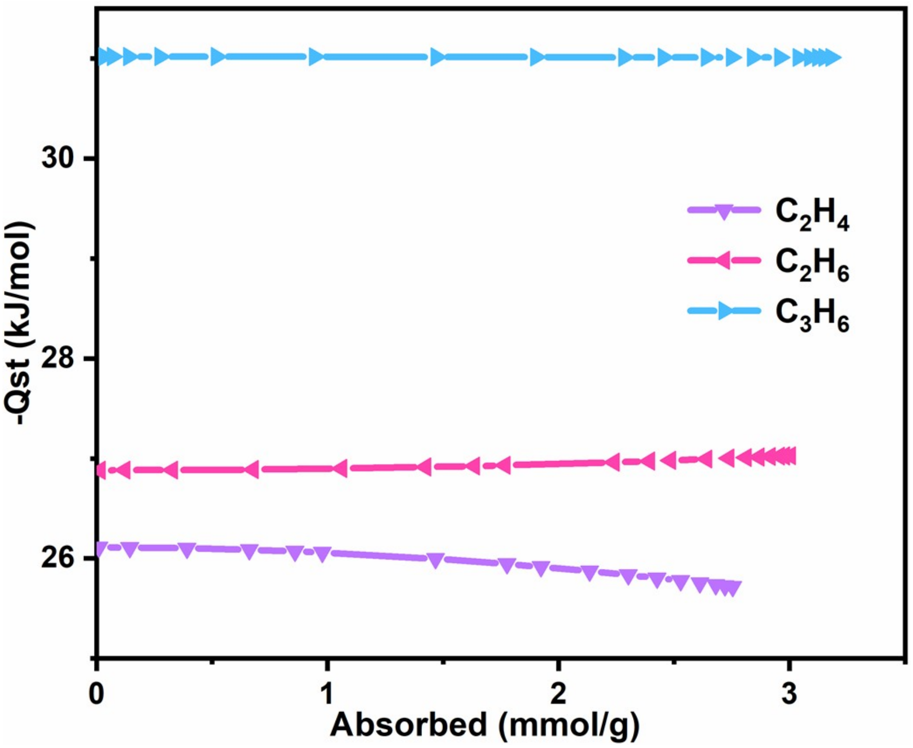

Fig. S10 The Qst curves of C_2H_4, C_2H_6 and C_3H_6.

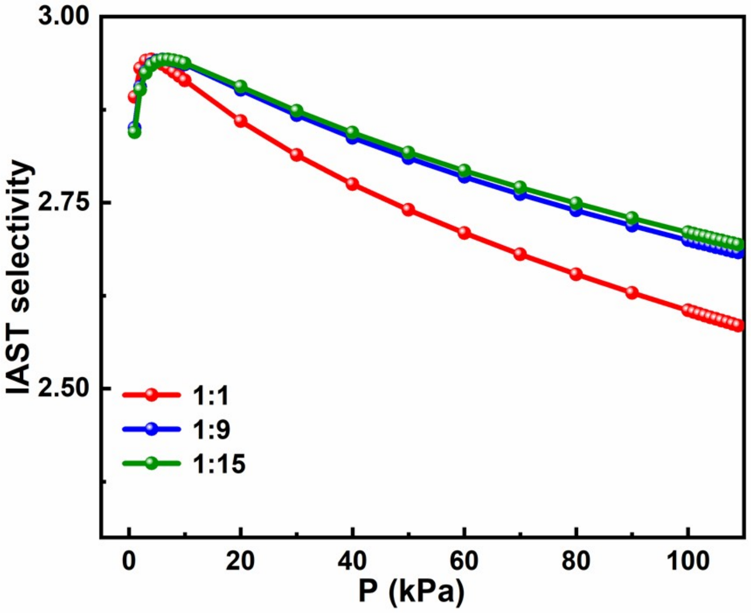

Fig. S11 IAST selectivity curves of Zn-hba for C_2H_6/C_2H_4 mixtures at 273K.

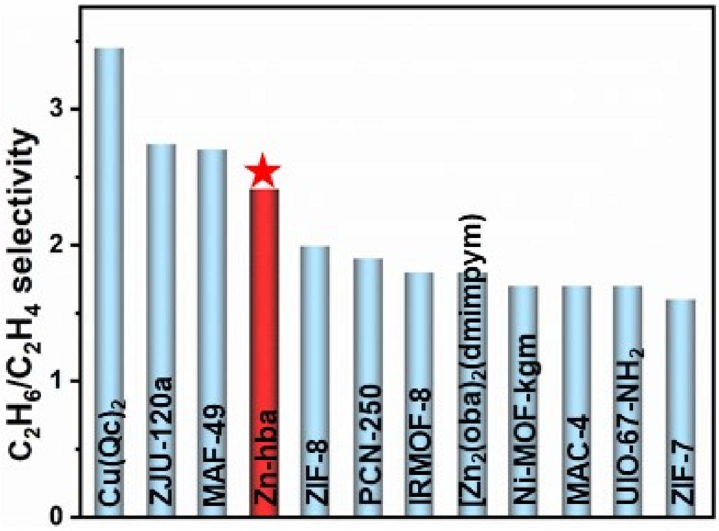

Fig. S12 Comparisons of the selectivity in Zn-hba and some benchmark materials for C_2H_6/C_2H_4 (1/1).

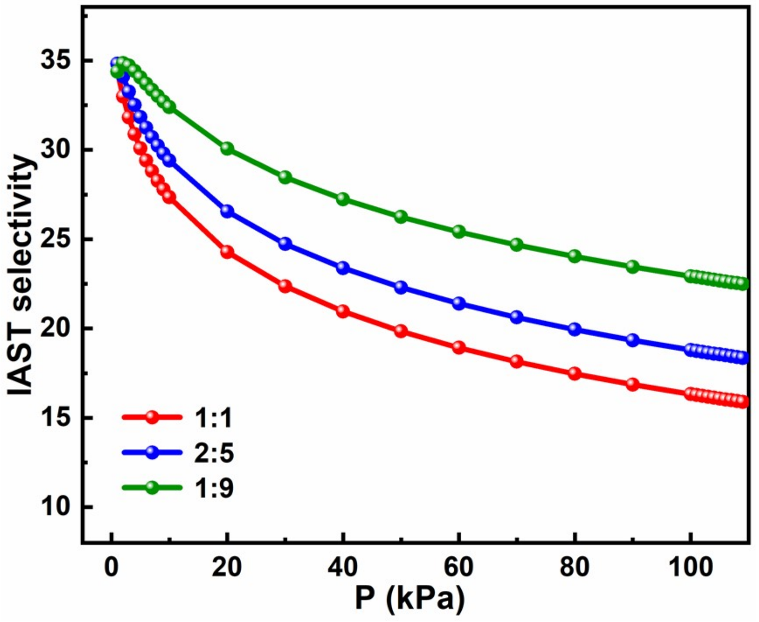

Fig. S13 IAST selectivity curves of Zn-hba for C_3H_6/C_2H_4 mixtures at 273K.

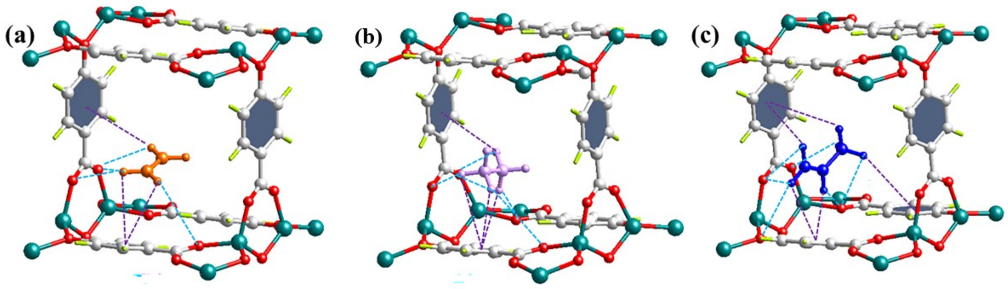

Fig. S14 The DFT-calculated configurations of (a) C_2H_4, (b) C_2H_6, and (c) C_3H_6 in Zn-hba.

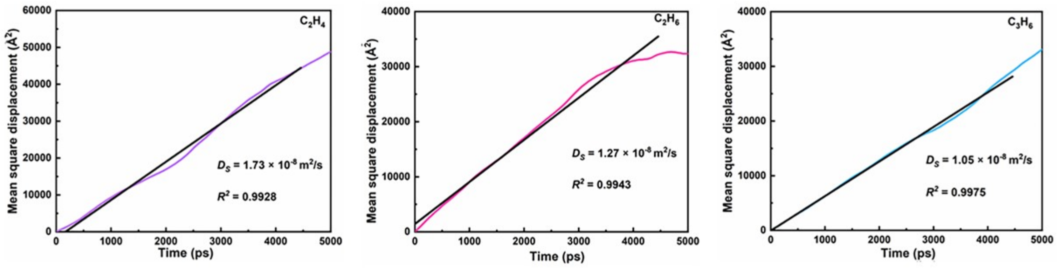

Fig. S15 MD-derived self-diffusion rate of C_2H_4, C_2H_6, and C_3H_6 in Zn-hba (black line represents linear fittings).

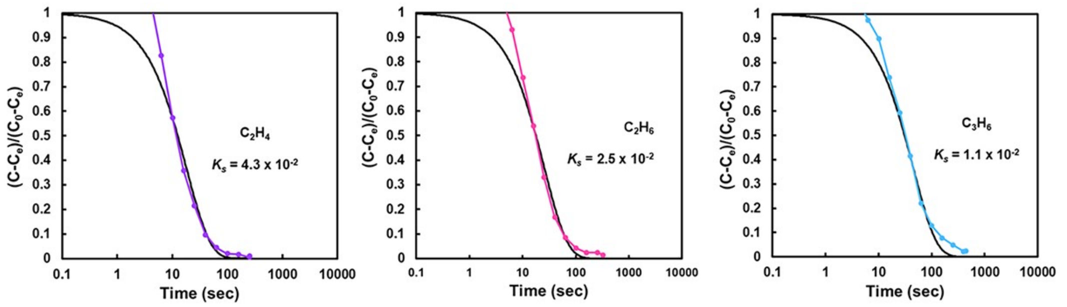

Fig. S16 The calculated diffusion coefficient of C_2H_4, C_2H_6, and C_3H_6 in Zn-hba based on kinetic experiments.

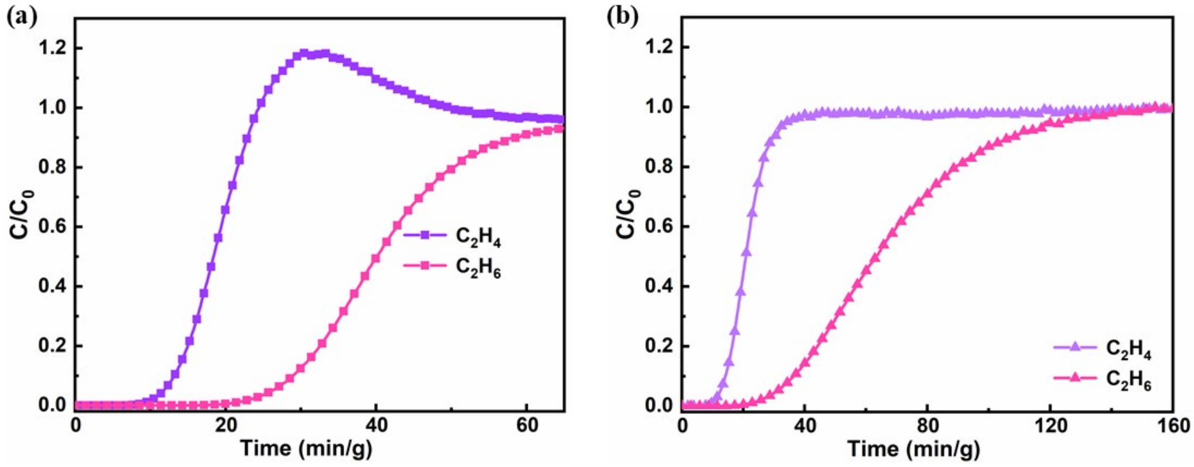

Fig. S17. Breakthrough curves for C_2H_6/C_2H_4/He (v/v, 5/5/90) (a) and (v/v, 1/ 9/ 90) (b) mixtures at 298 K with 10mL min^{-1} on large-scale synthesized samples.

.

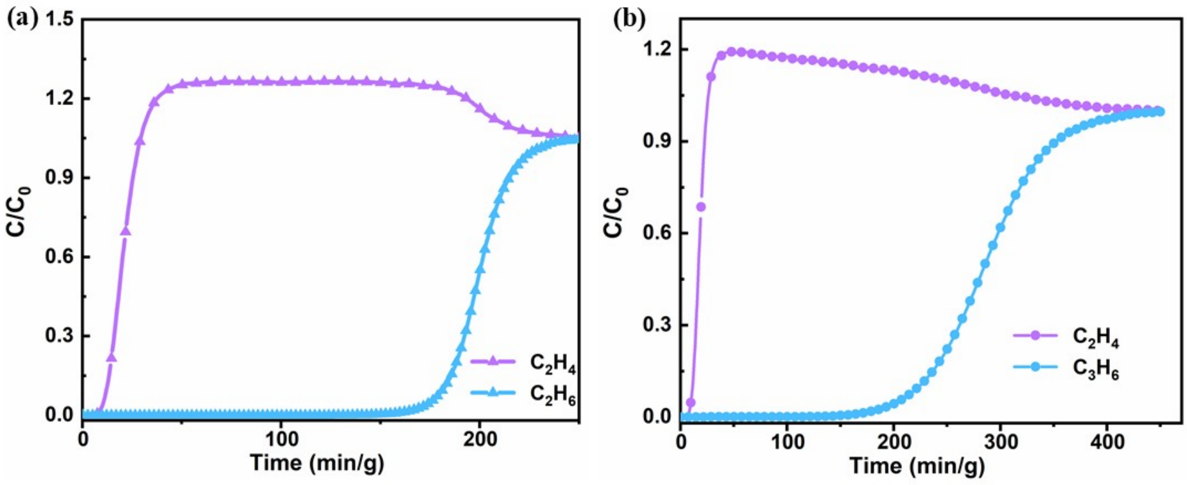

Fig. S18. Breakthrough curves for $C_3H_6/C_2H_4/He$ (v/v, 1/9/90) (a) and (v/v, 1/ 15 / 84) (b) mixtures at 298 K with 10mL min^{-1} on large-scale synthesized samples.

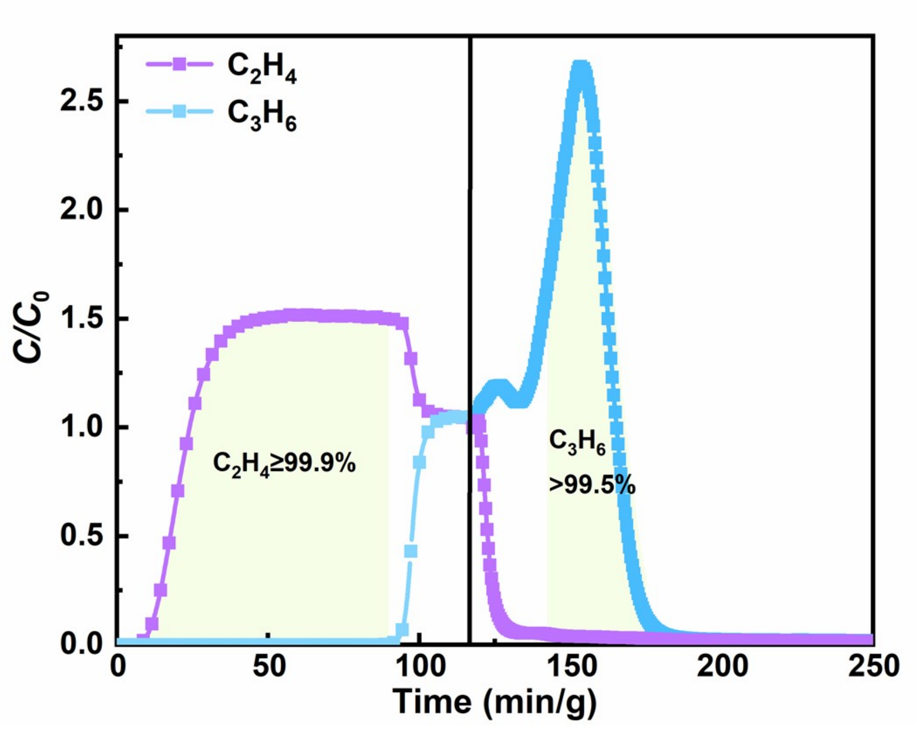

Fig. S19. Breakthrough curves for C_3H_6/C_2H_4 (v/v, 5/5) mixtures at 298 K on large-scale synthesized samples.

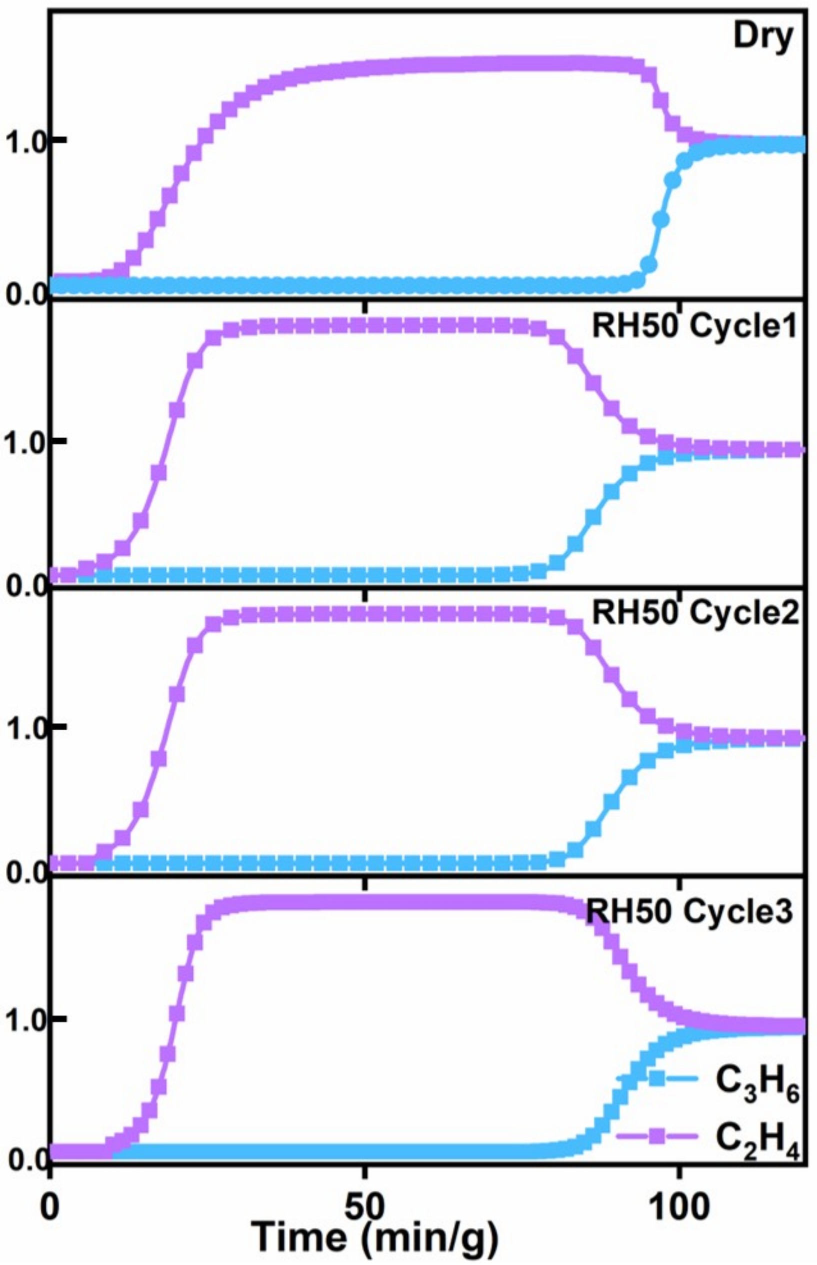

Fig. S20. Cycling breakthrough curves for 50/50 C_3H_6/C_2H_4 mixtures at 50% humid conditions at 298K on large-scale synthesized samples.

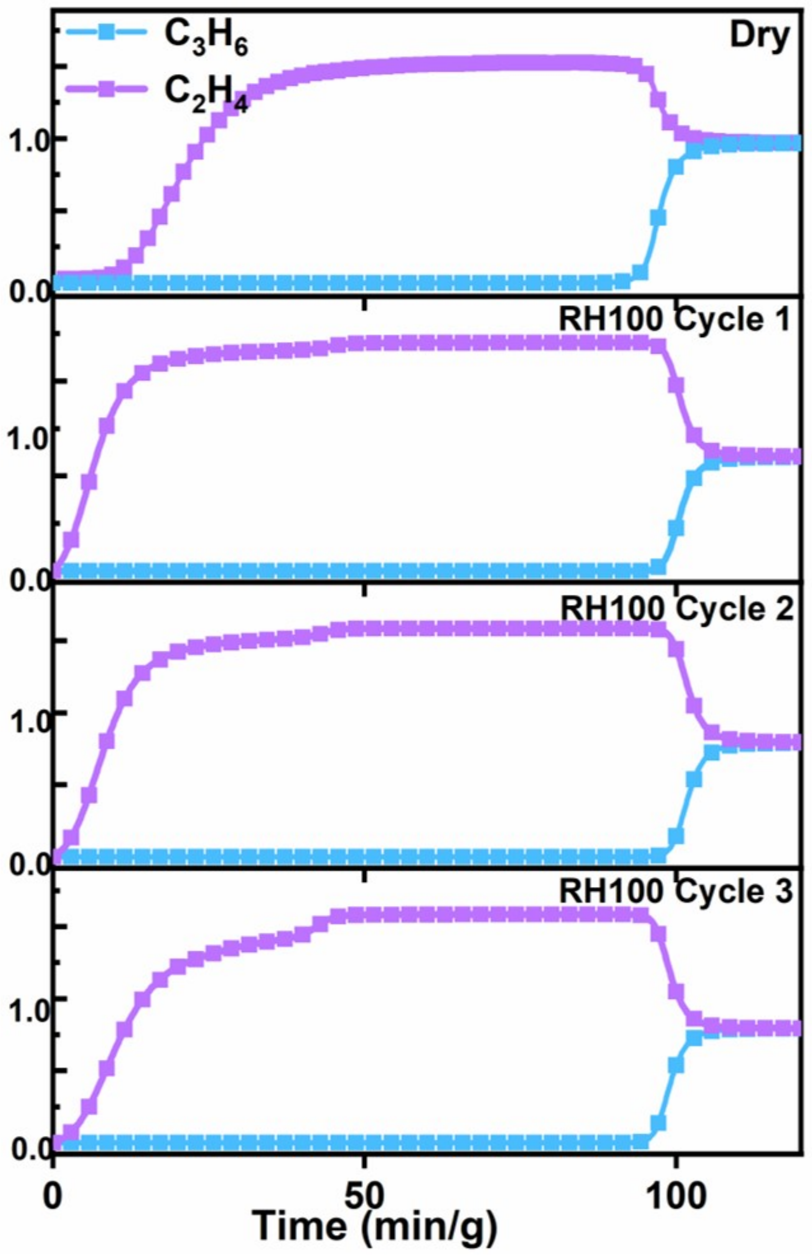

Fig. S21. Cycling breakthrough curves for 50/50 C_3H_6/C_2H_4 mixtures at 100% humid conditions at 298K on large-scale synthesized samples.

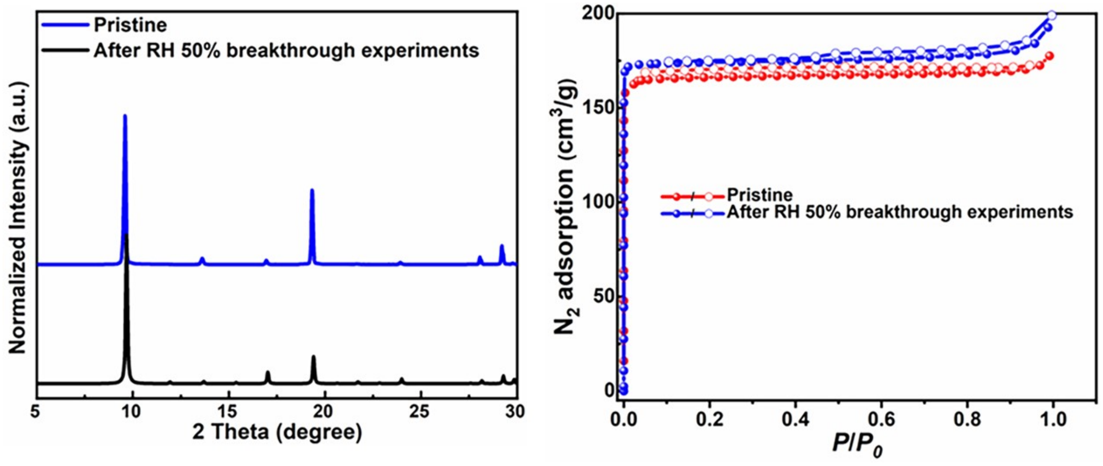

Fig. S22. The comparisons of PXRD and 77 K N_2 adsorption for pristine Zn-hba and the sample after 3 cycles of breakthrough experiments at 50% humid conditions.

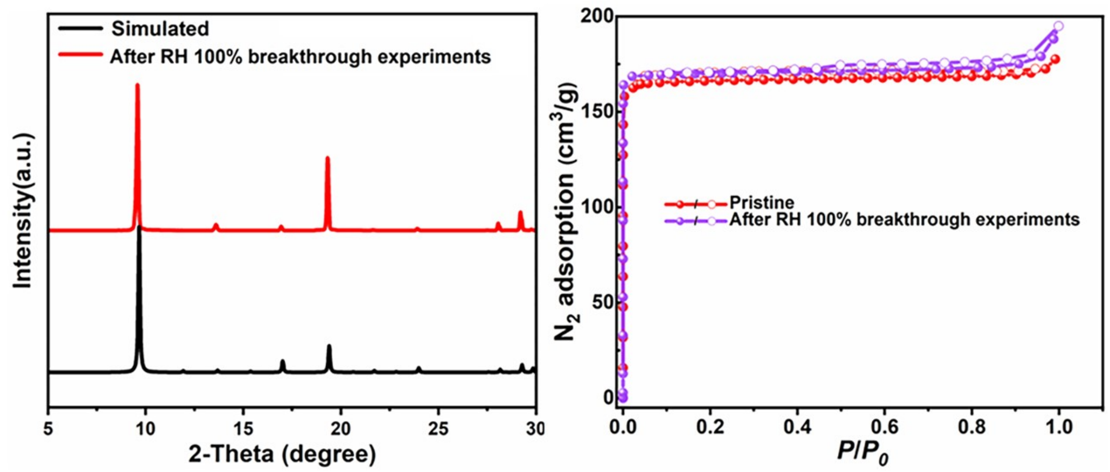

Fig. S23 The comparisons of PXRD and 77 K N_2 adsorption for pristine Zn-hba and the sample after 3 cycles of breakthrough experiments at 100% humid conditions.

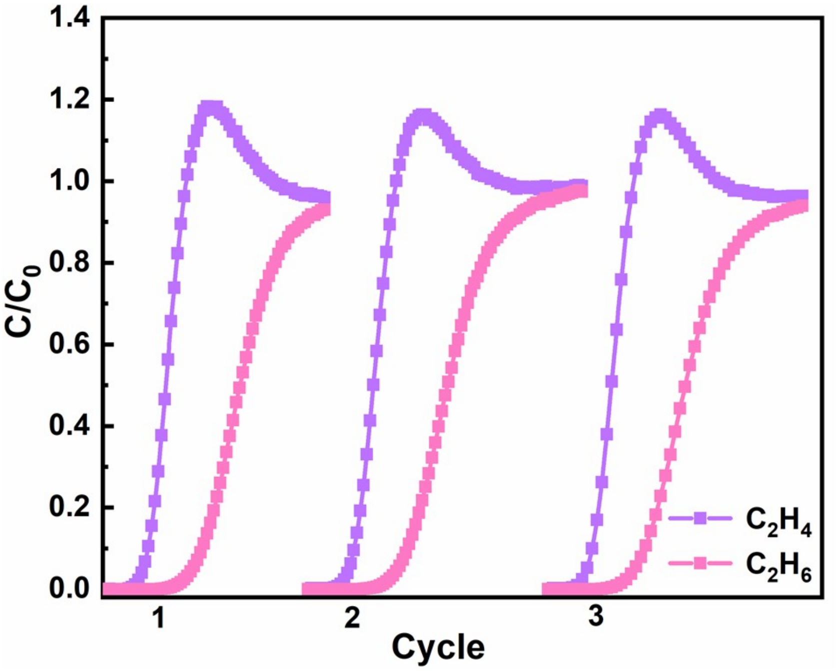

Fig. S24. Breakthrough cycles of Zn-hba for equimolar C_2H_6/C_2H_4 (v/v, 5/5) mixtures at 298K on large-scale synthesized samples.

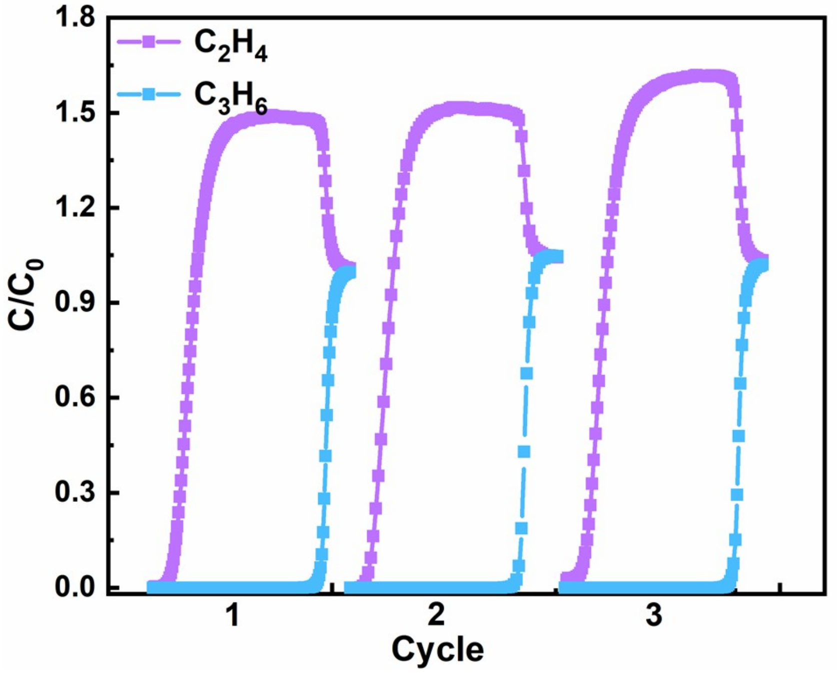

Fig. S25. Breakthrough cycles of Zn-hba for equimolar C_3H_6/C_2H_4 (v/v, 5/5) mixtures at 298K on large-scale synthesized samples.

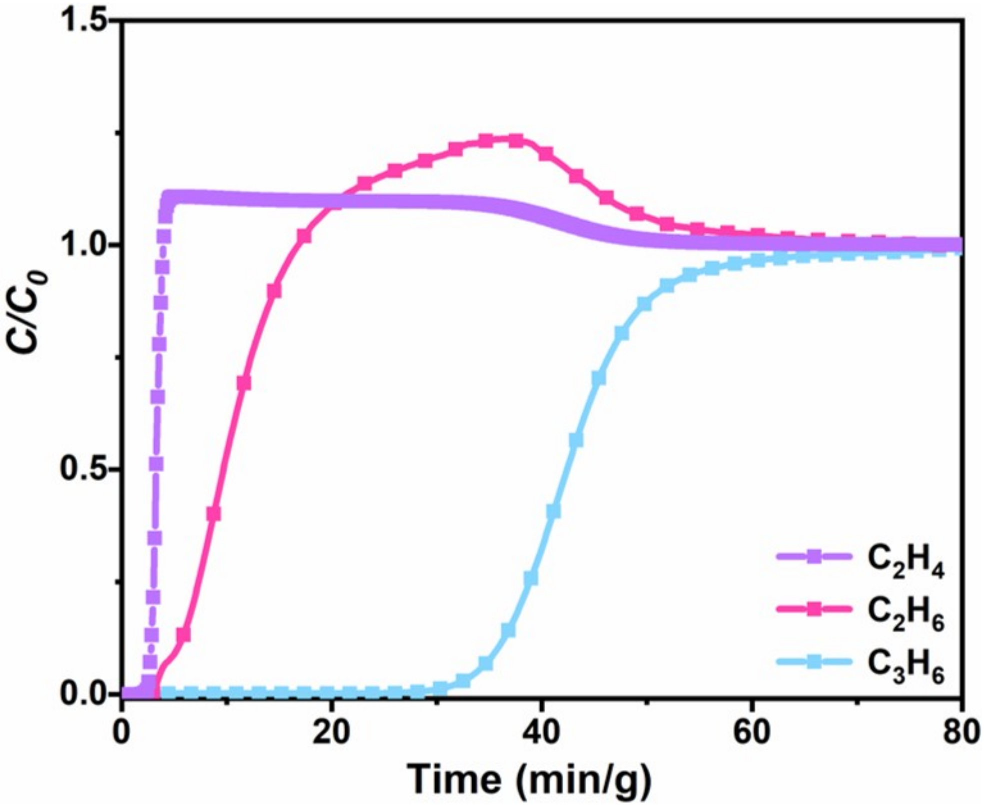

Fig. S26. Breakthrough curves of Zn-hba for the C_2H_6/C_3H_6/C_2H_4 mixture (v/v/v, 1/9/90) mixtures at 298K on large-scale synthesized samples.

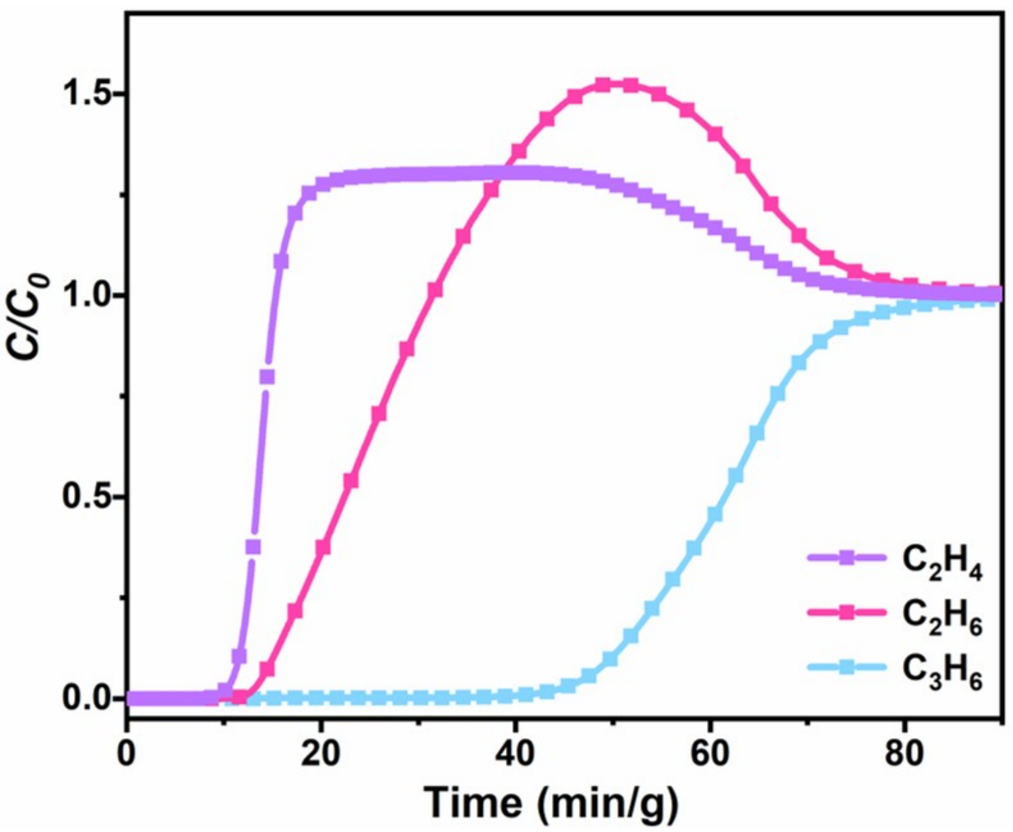

Fig. S27. Breakthrough curves of Zn-hba for the C_2H_6/C_3H_6/C_2H_4 mixture (v/v/v, 2/10/25) mixtures at 298K on large-scale synthesized samples.

Table S1. The raw material cost. The material cost estimation was exclusively based on the actual quantities of metal salts and organic ligand precursors utilized, while the cost of the operation and utility cost for hydrothermal reaction, filtrate, and drying, etc. are not considered.

Material	Price ($ Kg^{-1})	Source
$Zn(CH_3COO)_2 \cdot 2H_2O$	3.75 $ Kg^{-1}	Greagent
HBA	6.81 $ Kg^{-1}	Bidepharm
MEOH	2.58 $ L^{-1}	Greagent
n-Amyl alcohol	2.08 $ L^{-1}	Shandong Chuxin
Zn-hba	0.14 $ g^{-1}	

Table S2. Summary of adsorption capacity of C_3H_6 for different MOFs at 298 K under 1 kPa.

Sample	C_3H_6 uptake (cm^3/g)	Packing density (g/L)	Ref.
Zn-hba	38.9	257.5	This work
Al-TCPP	32.2	88.3	9
MAC-4	27.9	119.6	10
Zn-BPZ-TATB	19.3	69.1	11
[$Zn_2(oba)_2$(dmimpym)]	18.5	89.8	12
SIFSIX-Cu-TPA	16	52.8	13
Zn-BPZ-SA	9.7	36.2	14
FJI-Y9	9.6	36.3	15
Cd-dtzip-H_2O	9.1	35.2	16
Mn-dtzip	8.9	49.0	17
Cu-MeBPZ	6.0	15.9	18

Table S3. Summary of adsorption capacity of C_2H_6 for different MOFs at 298 K under 10 kPa.

Sample	C_2H_6 uptake (cm^3/g)	Packing density (g/L)	Ref.
NTU-70P	40.3	144.7	19
Zn-hba	39.5	186.9	This work
NUM-7a	34.8	219.7	20
MAC-4	33.3	101.9	10
Al-TCPP	32.5	62.6	8
CUPMOF-2-Mg	31.0	210.9	21
Zn-BPZ-TATB	28.9	73.7	10
CUPMOF-2-Cu	22.8	185.8	20
NTU-70D	22.3	56.6	18
Zn-FBA	21.5	228.5	22
ZNU-1	17.4	106.7	23
FJI-Y9	16.6	44.7	14
$[Zn_2(oba)_2(dmimpym)]$	16.2	56.0	11
CUPMOF-2-Zn	15.8	114.0	20
UIO-66	15.5	50.5	24
NTU-101-NH_2	13.1	144.7	25
NTU-101	13.0	120.7	24
CUPMOF-2-Mn	11.4	39.8	20
UIO-66-CF_3	10.7	48.5	23
ZNU-10	7.9	21.36	24
UPC-250	8.0	10.31	26
UPC-251	8.0	10.65	26
UIO-66-$(CF_3)_2$	5.7	29.16	23

References

1. K. F. White, B. F. Abrahams, R. Babarao, A. D. Dharma, T. A. Hudson, H. E. Maynard-Casely and R. Robson, A New Structural Family of Gas-Sorbing Coordination Polymers Derived from Phenolic Carboxylic Acids, *Chem. Eur. J.*, 2015, **21**, 18057-18061.
2. P. Hohenberg and W. Kohn, Inhomogeneous Electron Gas, *Phys. Rev.*, 1964, **136**, B864-B871.
3. W. Kohn and L. J. Sham, Self-Consistent Equations Including Exchange and Correlation Effects, *Phys. Rev.*, 1965, **140**, A1133-A1138.
4. J. VandeVondele, M. Krack, F. Mohamed, M. Parrinello, T. Chassaing and J. Hutter, Quickstep: Fast and accurate density functional calculations using a mixed Gaussian and plane waves approach, *Comput. Phys. Commun.*, 2005, **167**, 103-128.
5. S. Goedecker, M. Teter and J. Hutter, Separable dual-space Gaussian pseudopotentials, *Phys. Rev. B*, 1996, **54**, 1703-1710.
6. M. Krack and M. Parrinello, All-electron ab-initio molecular dynamics, *PCCP*, 2000, **2**, 2105-2112.
7. J. P. Perdew, K. Burke and M. Ernzerhof, Generalized Gradient Approximation Made Simple, *Phys. Rev. Lett.*, 1996, **77**, 3865-3868.
8. P. Hu, J. Han, J. Zhou, H. Wang, C. Xiong, H. Liu, X. Zhou, Y. Wang and H. Ji, Customized H-bonding acceptor and aperture chemistry within a metal-organic framework for efficient C3H6/C3H8 separation, *Chem. Eng. J.*, 2021, **426**, 131302.
9. J. Xiao, Z. Zhu, M. Zhang, Y. Huang, T. C. Zhang and S. Yuan, Efficient One-Step Purification of Methanol-to-Olefin Products Using a Porphyrinyl MOF to Achieve Record C2H4 and C3H6 Productivity, *ACS Appl. Mater. Interfaces*, 2025, **17**, 21630-21642.
10. G.-D. Wang, Y.-Z. Li, R. Krishna, W.-Y. Zhang, L. Hou, Y.-Y. Wang and Z. Zhu, Scalable Synthesis of Robust MOF for Challenging Ethylene Purification and Propylene Recovery with Record Productivity, *Angew Chem. Int. Ed.*, 2024, **63**, e202319978.
11. G.-D. Wang, Y.-Z. Li, W.-J. Shi, L. Hou, Y.-Y. Wang and Z. Zhu, Active Sites Decorated Nonpolar Pore-Based MOF for One-step Acquisition of C2H4 and Recovery of C3H6, *Angew Chem. Int. Ed.*, 2023, **62**, e202311654.
12. Y.-Z. Li, G.-D. Wang, R. Krishna, Q. Yin, D. Zhao, J. Qi, Y. Sui and L. Hou, A separation MOF with O/N active sites in nonpolar pore for One-step C2H4 purification from C2H6 or C3H6 mixtures, *Chem. Eng. J.*, 2023, **466**, 143056.
13. Z. Ji, Y. Zhou, Y. Zhu, Y. Liu, Z. Di, M. Hong and M. Wu, A Highly Stable Anion-Functionalized Cage-like Framework for Efficient Separation of MTO Products under Harsh Conditions, *ACS Materials Lett.*, 2025, **7**, 837-844.
14. G.-D. Wang, R. Krishna, Y.-Z. Li, Y.-Y. Ma, L. Hou, Y.-Y. Wang and Z. Zhu, Rational Construction of Ultrahigh Thermal Stable MOF for Efficient Separation of MTO Products and Natural Gas, *ACS Materials Lett.*, 2023, **5**, 1091-1099.
15. H. Zhao, S. Guo, X. Chen, J. Jiang, S. Wang, H. Zhang, Y. Wang, X. He, M. Chen, W. Wang, S. Wang, P. Liu, H. Dai and M. Zhang, Flow Channel with Wrinkles and Calcium Sites in a Ca-MOF for Direct One-Step Ethylene Purification from C2 Gases and MTO Products Separation, *Inorg. Chem.*, 2024, **63**, 7113-7117.
16. L. Zhang, R.-C. Gao, X. Liu, B. Zhang, L. Hou and Y.-Y. Wang, Unique H2O Vortex in One New MOF Material Strengthening C3H6 Adsorption and Unprecedented Purification from C2H4/C3H8/C3H6 Mixtures, *Adv. Funct. Mater.*, 2025, **35**, 2420927.
17. L. Zhang, L.-N. Ma, G.-D. Wang, L. Hou, Z. Zhu and Y.-Y. Wang, A new honeycomb MOF for C2H4 purification and C3H6 enrichment by separating methanol to olefin products, *J. Mater. Chem. A*, 2023, **11**, 2343-2348.
18. G. Zhen, Y. Liu, Y. Zhou, Z. Ji, H. Li, S. Zou, W. Zhang, Y. Li, Y. Liu, C. Chen and M. Wu, Water-Stable Microporous Bipyrazole-Based Framework for Efficient Separation of

MTO Products, *ACS Appl. Mater. Interfaces*, 2024, **16**, 1179-1186.
19. J. Tang, Q. Wang, H. Zhang, Y. Huang, J. Duan and W. Jin, Repairing Lattice Defects by an Orienting Strategy in a Porous Crystal: Boosting Inverse C2H6/C2H4 Separation, *Small*, 2025, **21**, 2412508.
20. F.-Z. Sun, S.-Q. Yang, R. Krishna, Y.-H. Zhang, Y.-P. Xia and T.-L. Hu, Microporous Metal–Organic Framework with a Completely Reversed Adsorption Relationship for C2 Hydrocarbons at Room Temperature, *ACS Appl. Mater. Interfaces*, 2020, **12**, 6105-6111.
21. X.-C. Wu, L. Zhao, Y.-J. Tian, J.-S. Zou, Y. Jia, Z.-Y. Zhang, J.-H. Li, Y.-L. Peng, E. Yao and G. Chen, A large-scale synthesizable superhydrophobic C2H6- selective MOF for C2H6/C2H4 separation, *Micropor. Mesopor. Mat.*, 2024, **378**, 113257.
22. L. Yang, L. Yan, W. Niu, Y. Feng, Q. Fu, S. Zhang, Y. Zhang, L. Li, X. Gu, P. Dai, D. Liu, Q. Zheng and X. Zhao, Adsorption in Reversed Order of C2 Hydrocarbons on an Ultramicroporous Fluorinated Metal-Organic Framework, *Angew Chem. Int. Ed.*, 2022, **61**, e202204046.
23. L. Wang, S. Wu, J. Hu, Y. Jiang, J. Li, Y. Hu, Y. Han, T. Ben, B. Chen and Y. Zhang, A novel hydrophobic carborane-hybrid microporous material for reversed C2H6 adsorption and efficient C2H4/C2H6 separation under humid conditions, *Chem. Sci.*, 2024, **15**, 5653-5659.
24. J. Liu, J. Miao, H. Wang, Y. Gai and J. Li, Enhanced one-step purification of CH from CH/CH/CH mixtures by fluorinated Zr-MOF, *AIChE Journal*, 2023, **69**, e18021.
25. W. Yang, J. Wang, K. Tan, H.-L. Zhou, M. Zhang, R. Krishna, J. Duan and L. Huang, Regulating the Dynamics of Interpenetrated Porous Frameworks for Inverse C2H6/C2H4 Separation at Elevated Temperature, *Angew Chem. Int. Ed.*, 2025, **64**, e202425638.
26. M. Xu, Y. Li, X. Wang, H. Liu, Q. Liu, Y. Zhang, W. Fan, Q. Meng and D. Sun, Imidazole-Functionalized Zn-MOFs for One-Step C2H4 Purification from C2H2/C2H4/C2H6 Ternary Mixture, *Inorg. Chem.*, 2025, **64**, 813-817.
